# Supplementary material for: Variation in genetics, morphology, and recruitment of the invasive barnacle Amphibalanus eburneus (Gould, 1841) in the southern Korean peninsula
Source: PeerJ. 2022 Sep 2;10:e14002. doi: 10.7717/peerj.14002 (PMC9443810; doi:10.7717/peerj.14002)
Supplement: Supplemental Information 1 [file peerj-10-14002-s001.docx]

| Locality | May 2020 | Jul 2020 | Oct 2020 | Jan 2021 | April 2021 |
| --- | --- | --- | --- | --- | --- |
| Temperature (℃) | | | | | |
| Incheon | 14.9 | 24.3 | 21.1 | 5.2 | 11.9 |
| Tongyeong | 16.3 | 20.9 | 20.5 | 7.3 | 15.9 |
| Sokcho | 15.7 | 21.5 | 17.6 | 6.2 | 11.3 |
| Hanlim | 17.68 | 21.9 | 21.5 | 11.2 | 17.8 |
| Salinity (ppt) | | | | | |
| Incheon | 30.1 | 29.7 | 25.9 | 28.2 | 29 |
| Tongyeong | 34 | 31.2 | 31.7 | 33.5 | 33.7 |
| Sokcho | 34.1 | 29.16 | 32.7 | 33.1 | 34.2 |
| Hanlim | 35.6 | 32.1 | 31.5 | 31.2 | 34.3 |
