# Supplementary figures and images for: Variation in genetics, morphology, and recruitment of the invasive barnacle Amphibalanus eburneus (Gould, 1841) in the southern Korean peninsula
Source: PeerJ. 2022 Sep 2;10:e14002. doi: 10.7717/peerj.14002 (PMC9443810; doi:10.7717/peerj.14002)

# 1. Incheon

(1)

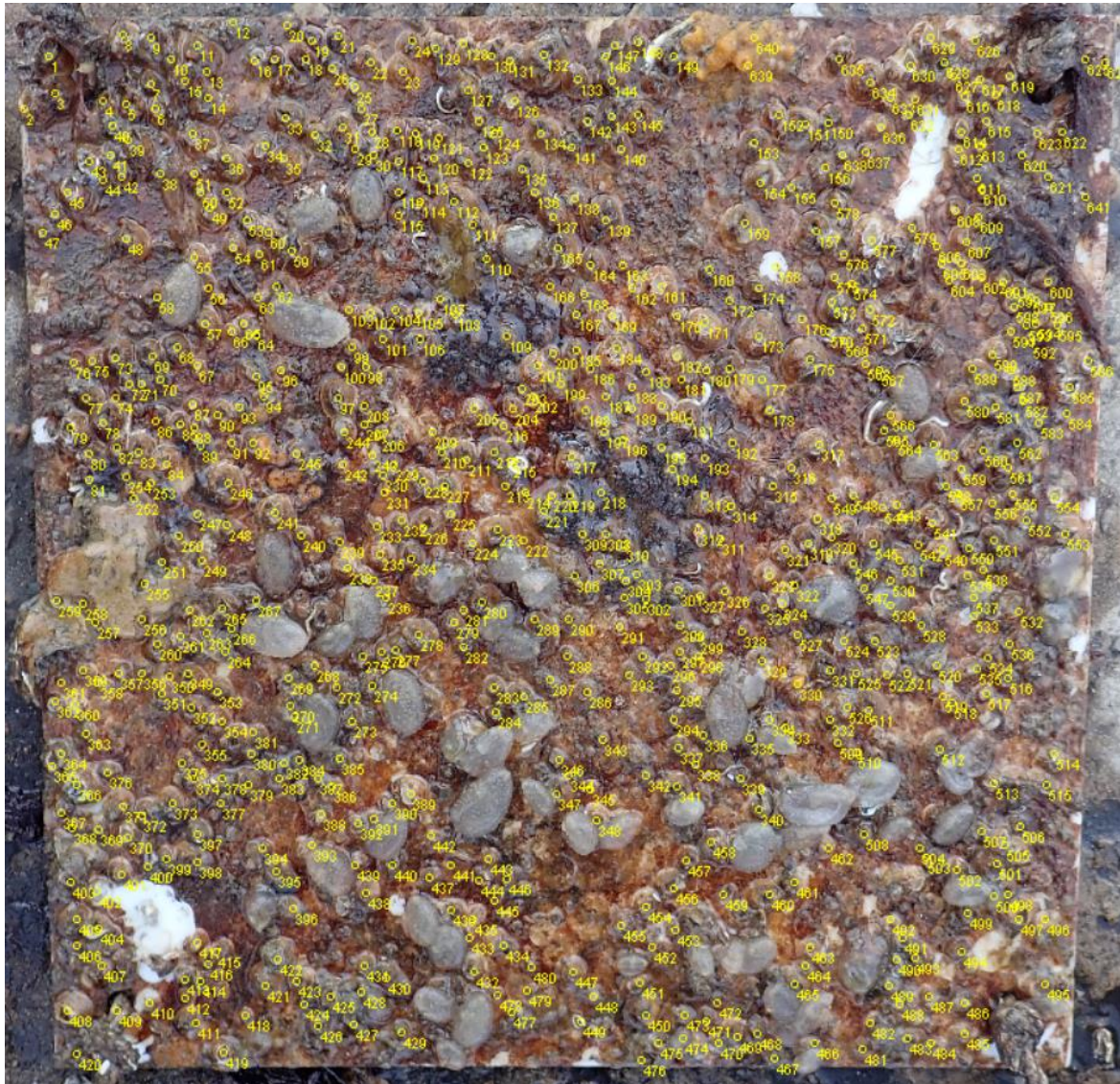

(2)

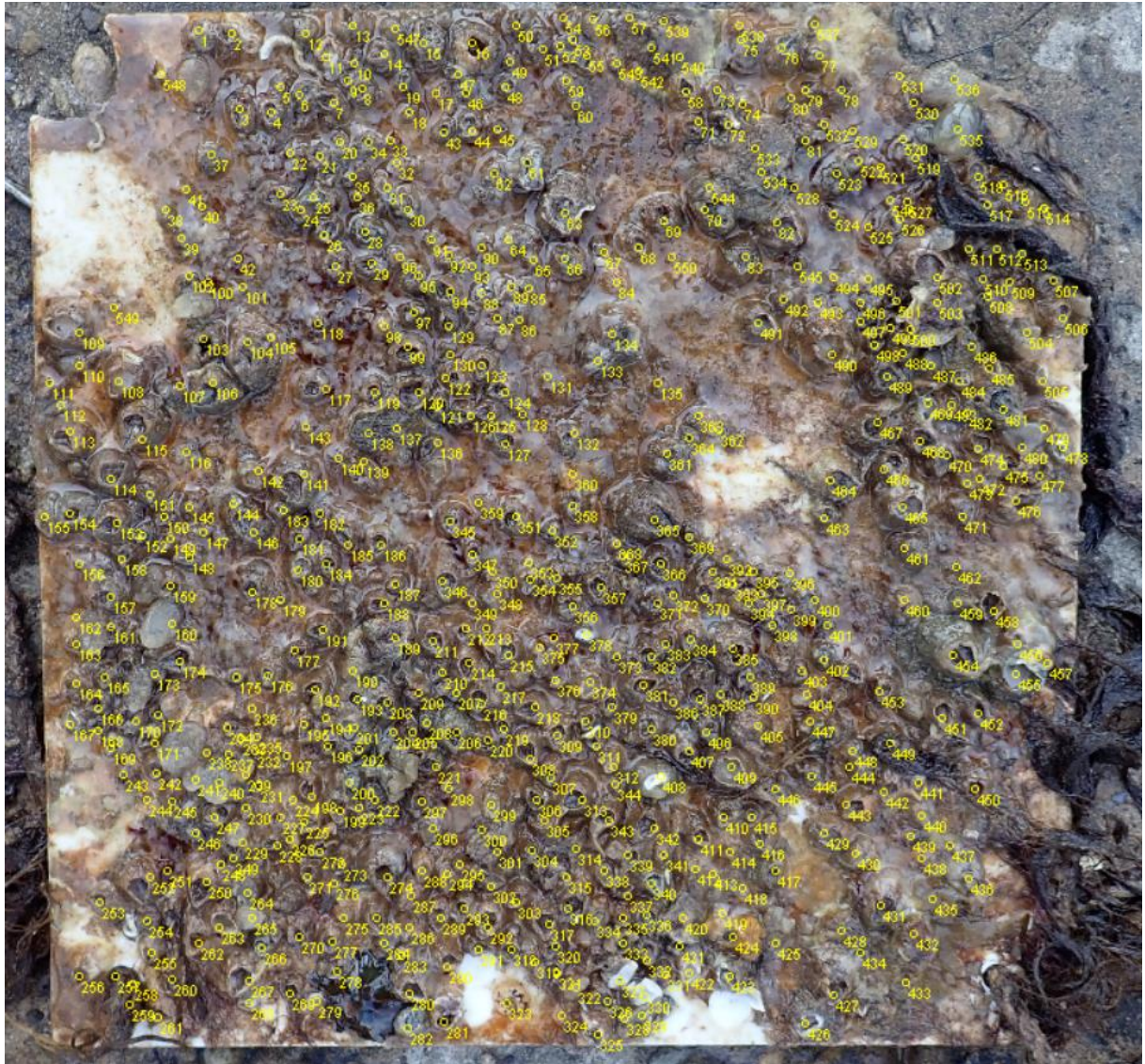

(3)

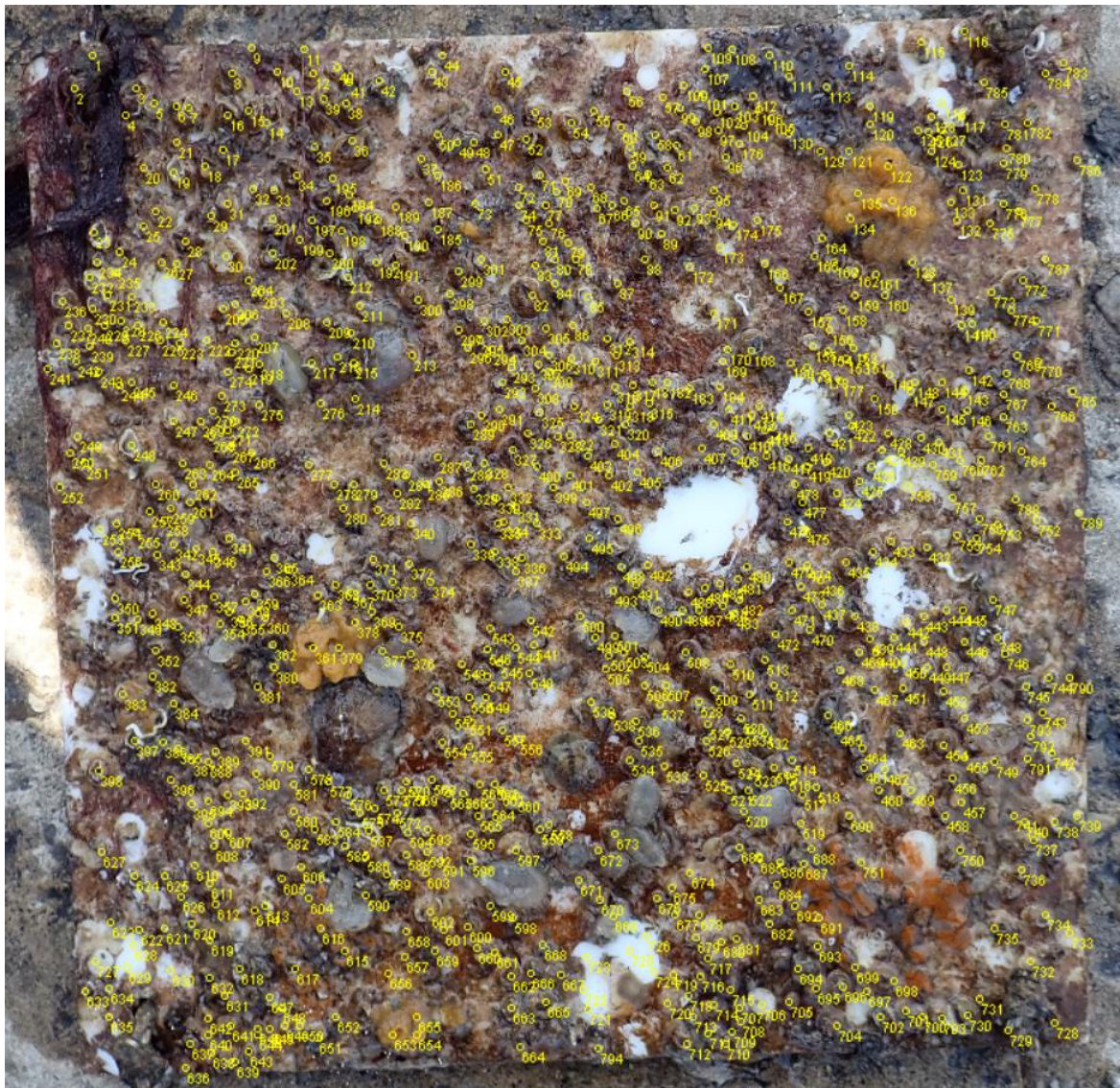

(4)

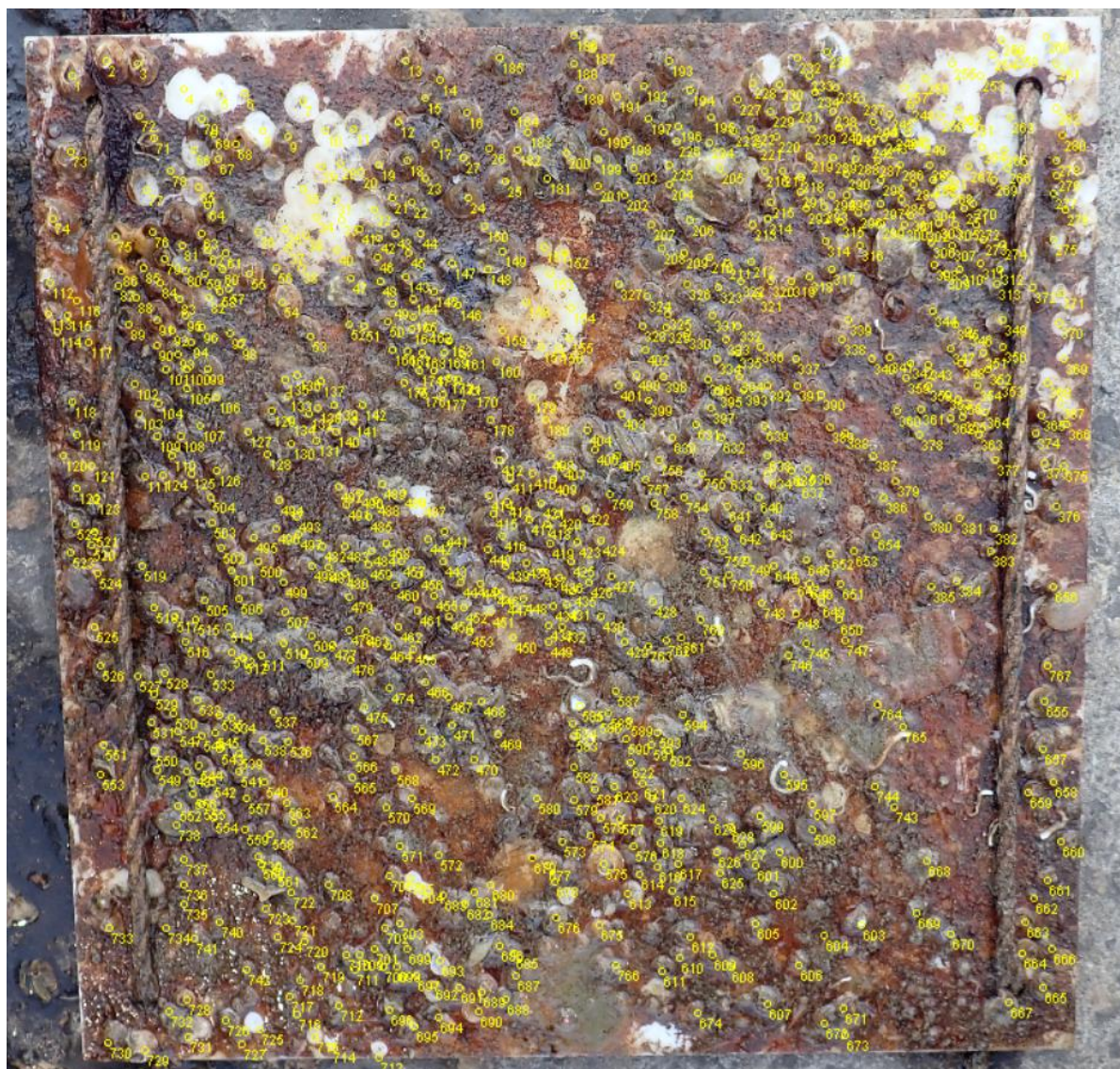

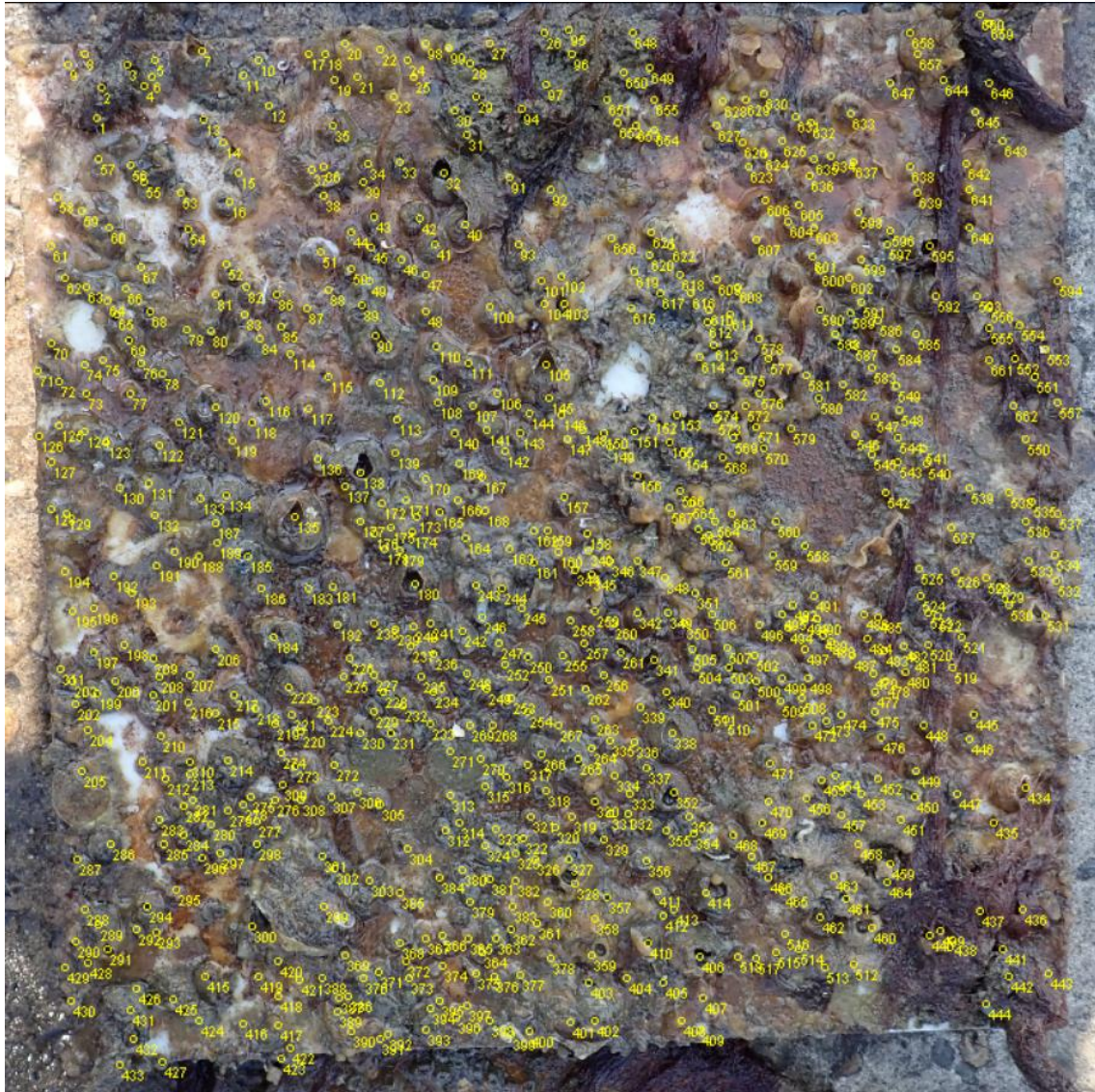

(5)

(6)

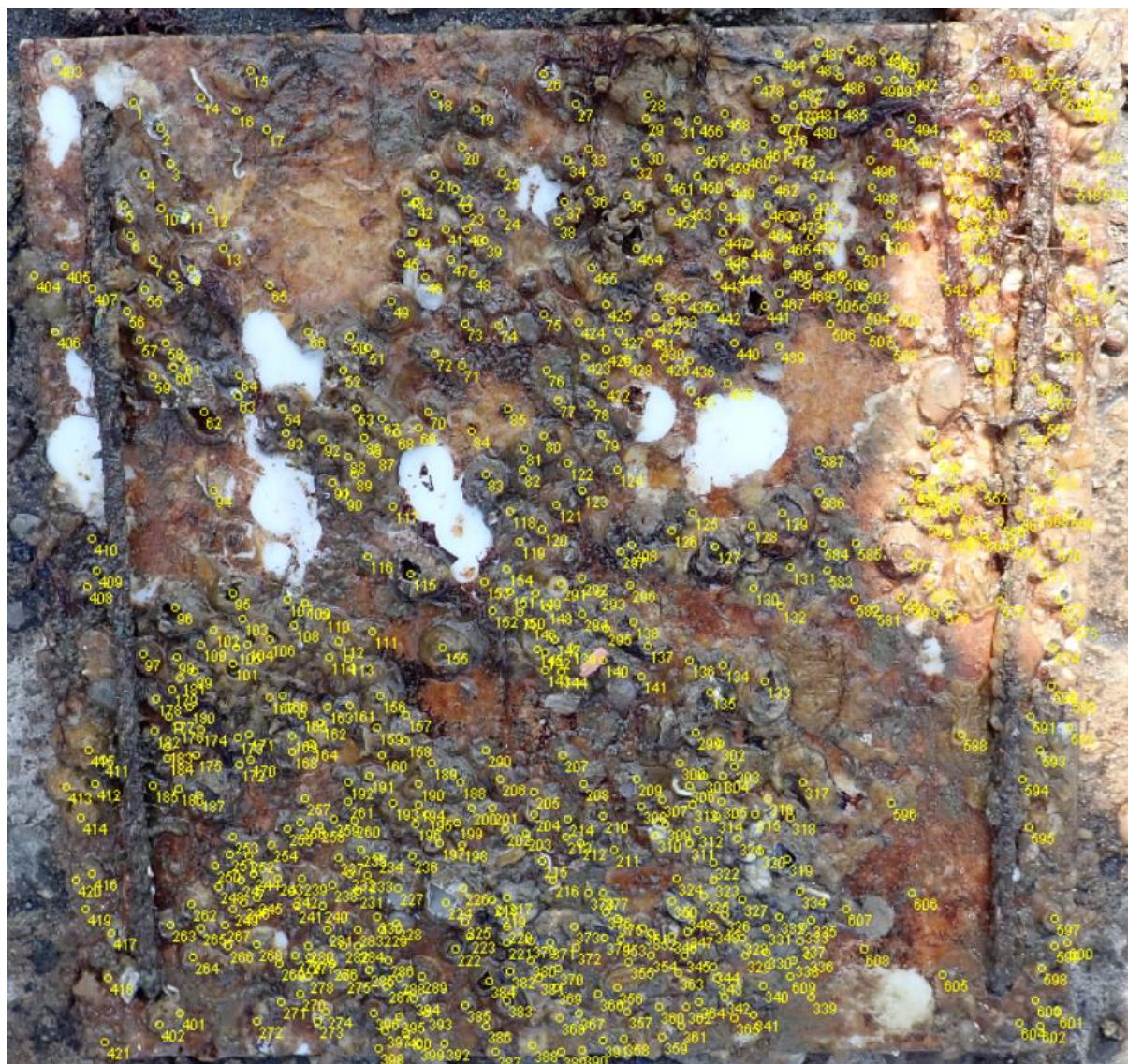

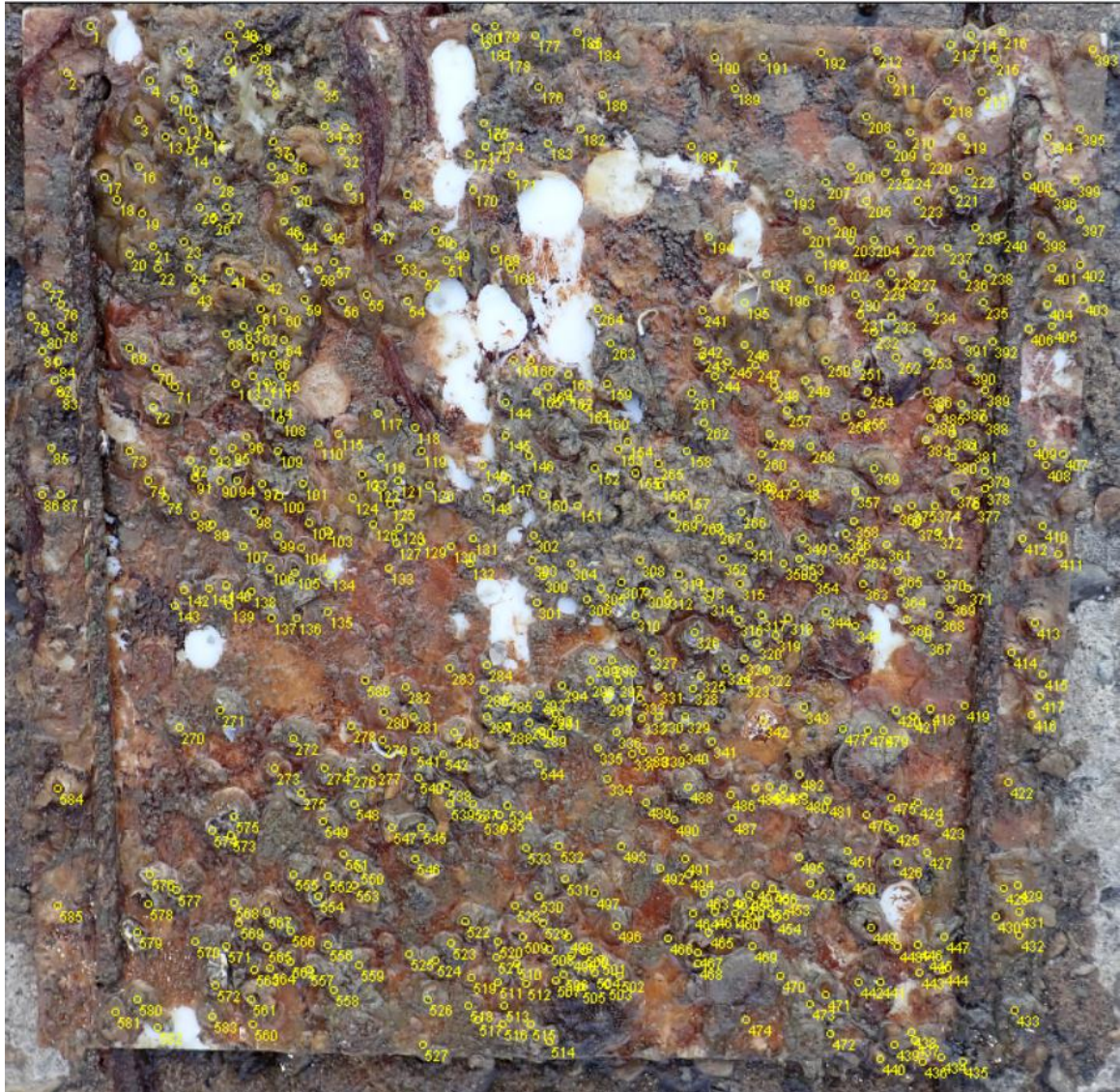

(7)

(8)

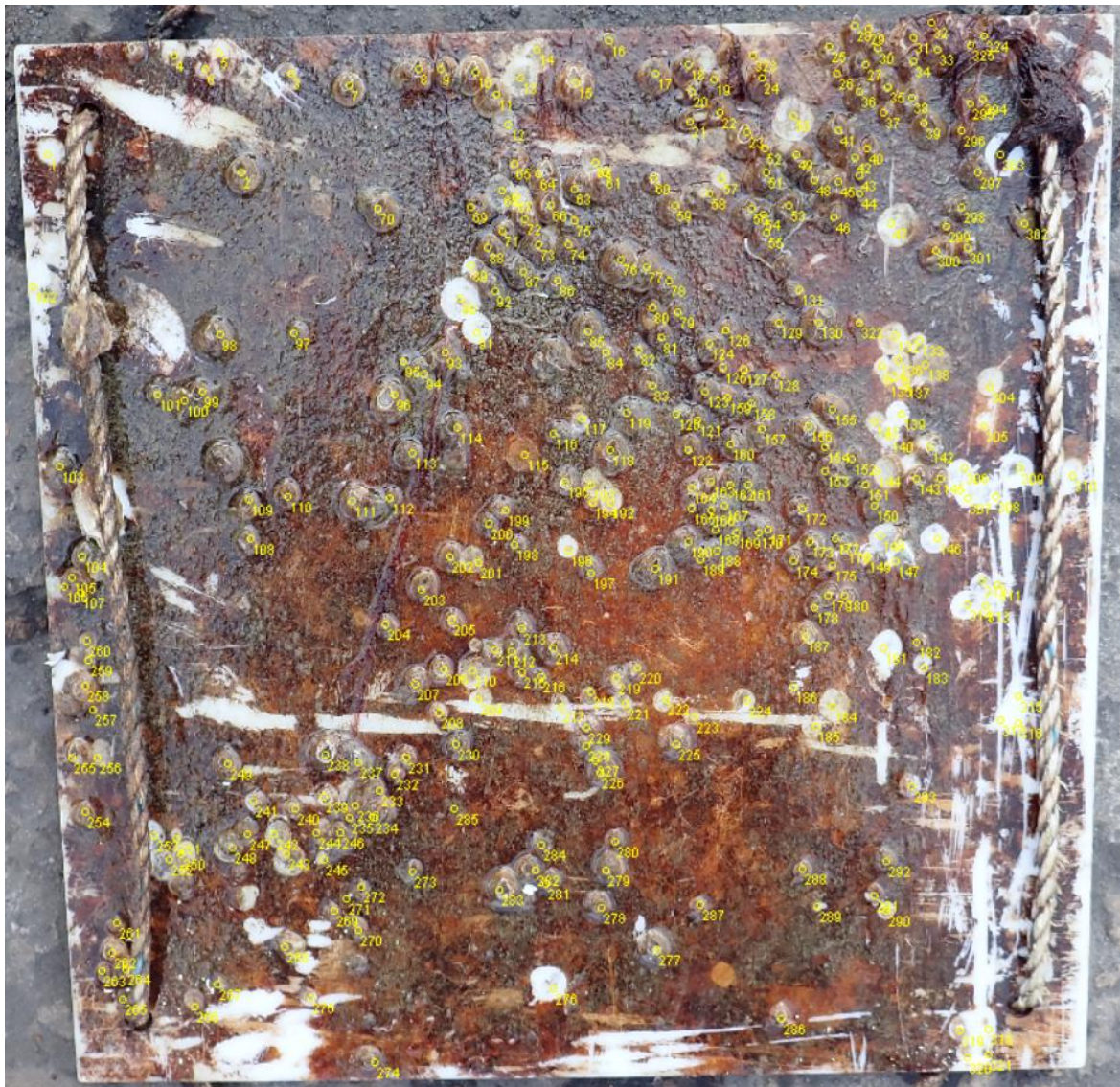

(9)

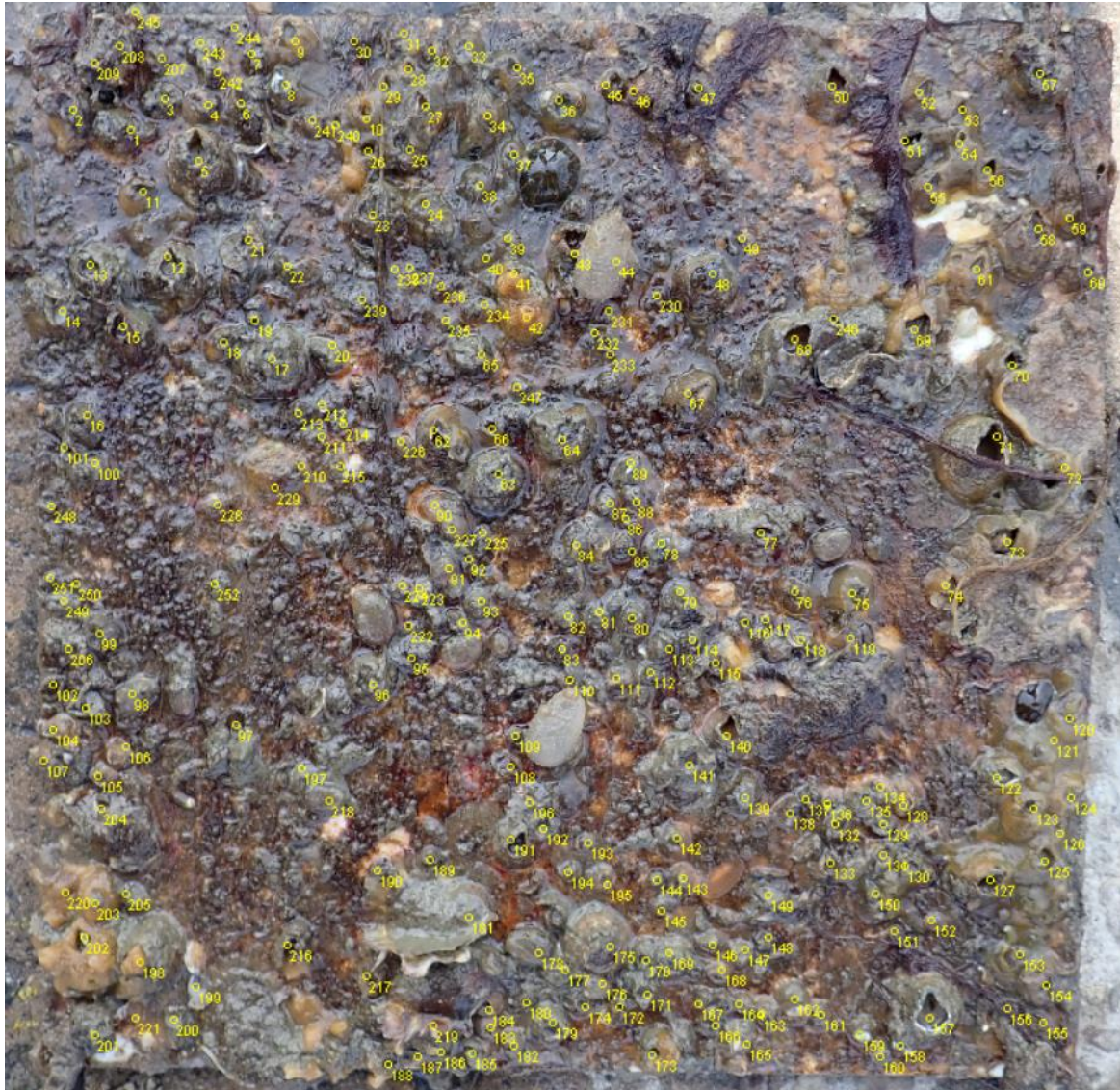

(10)

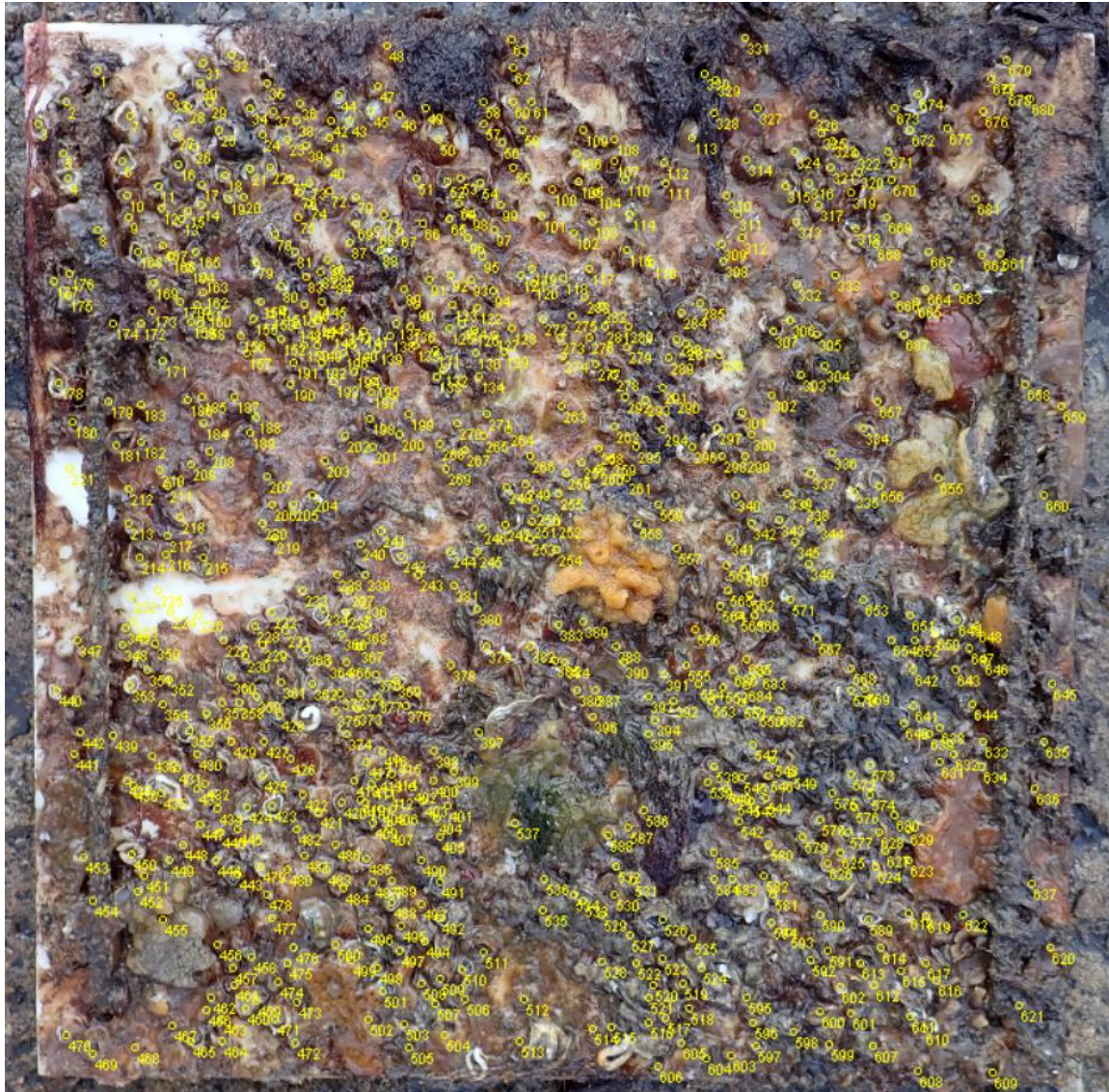

## 2. Tongyeong

(1)

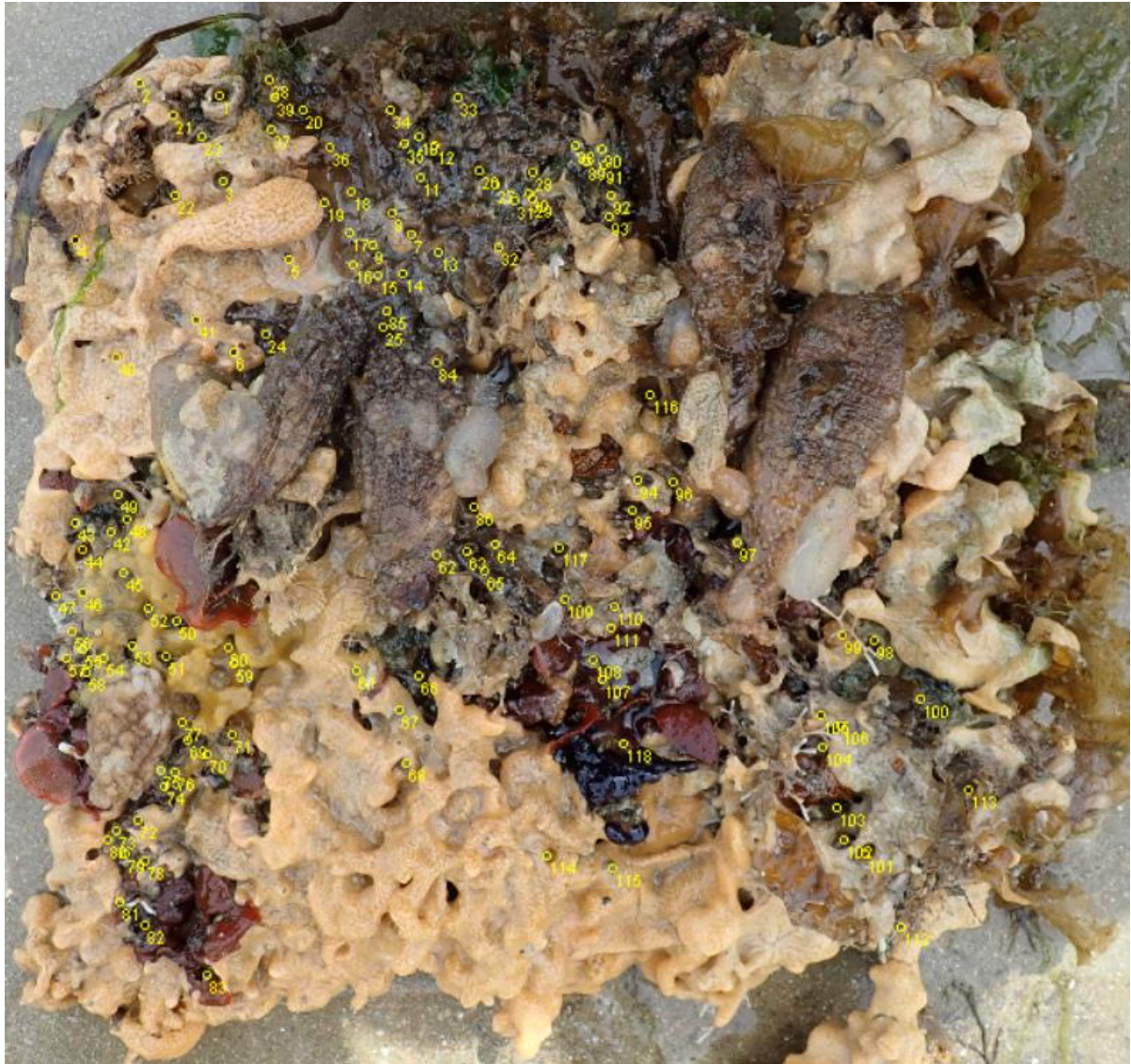

(2)

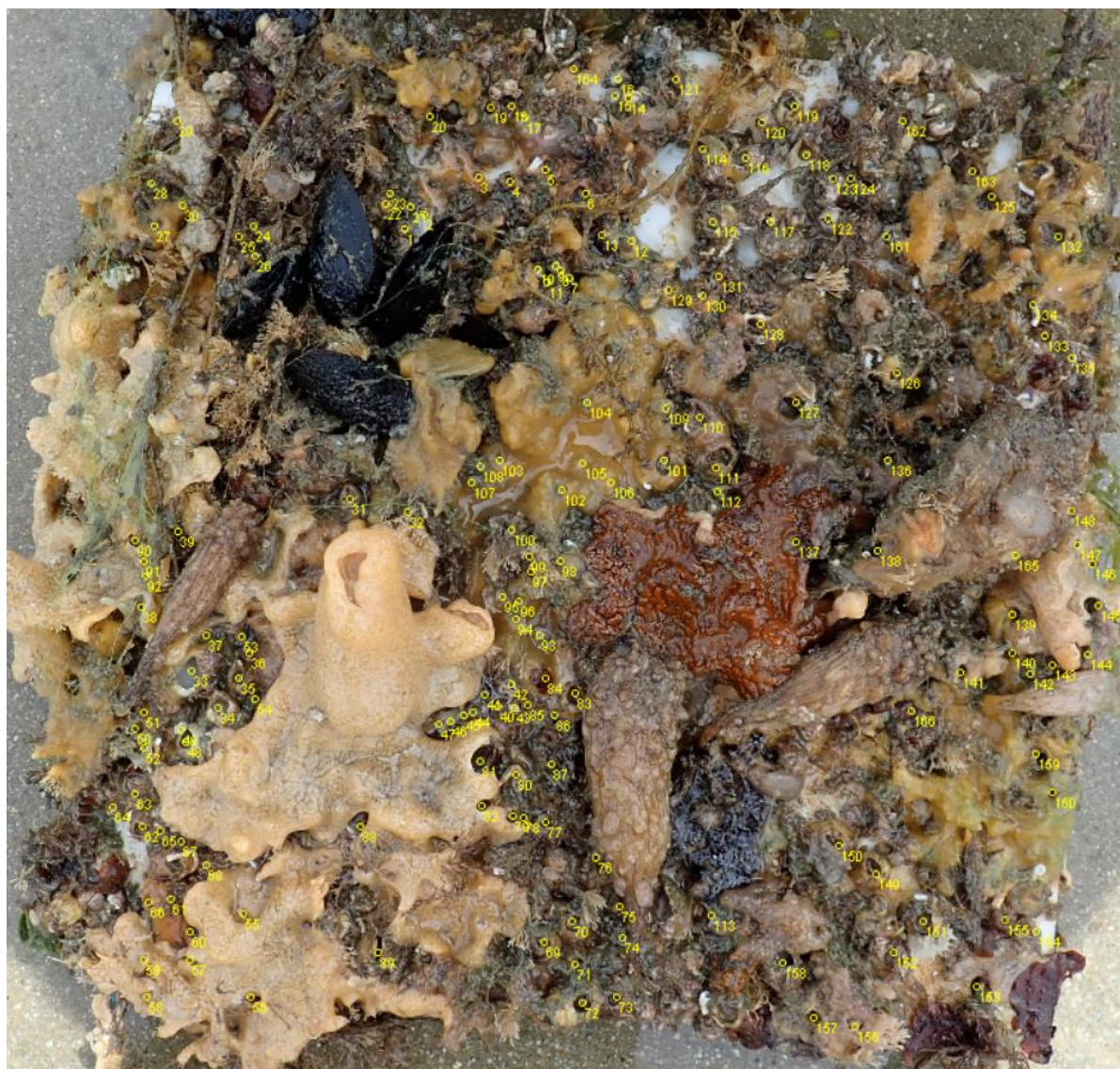

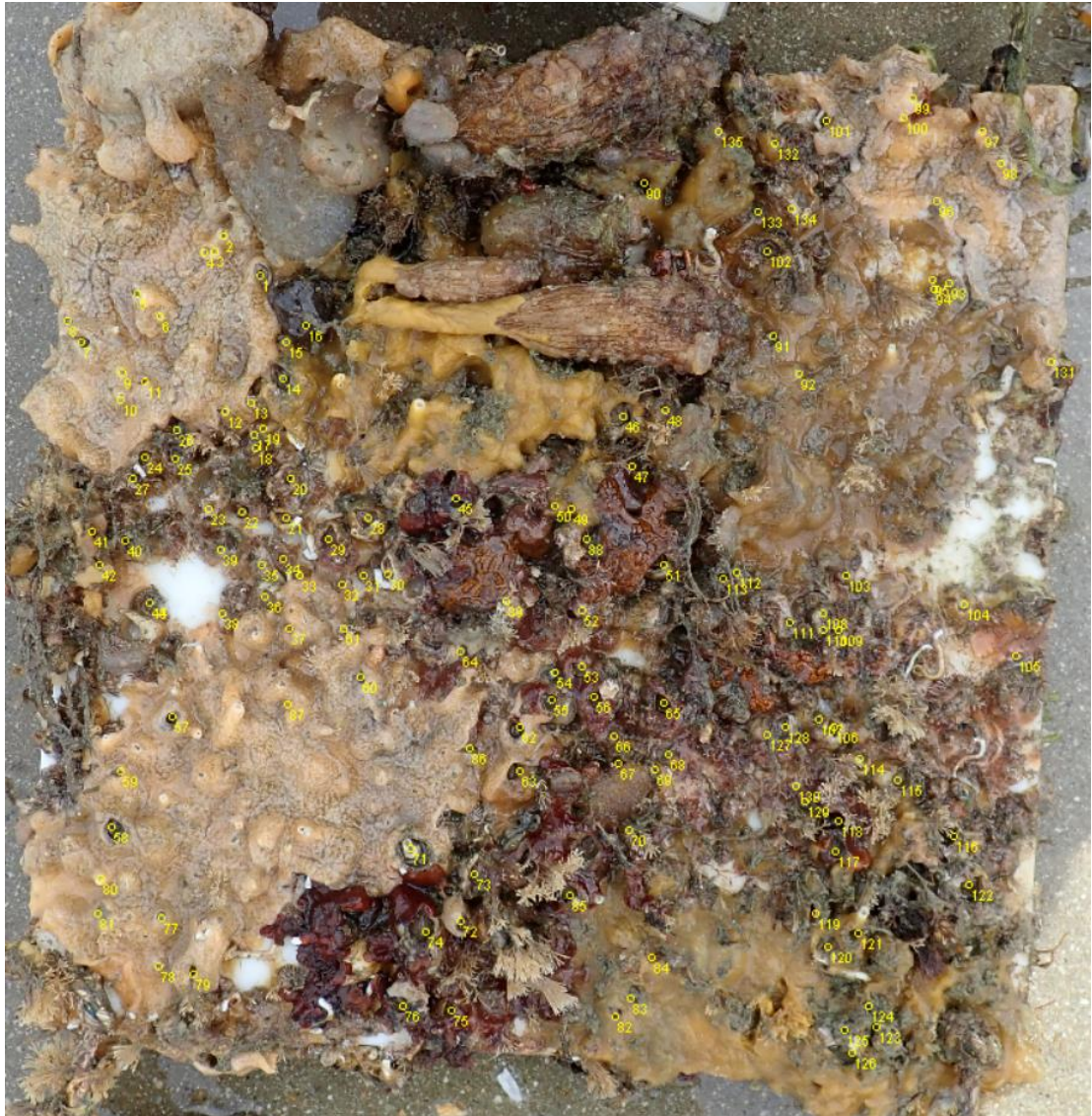

(3)

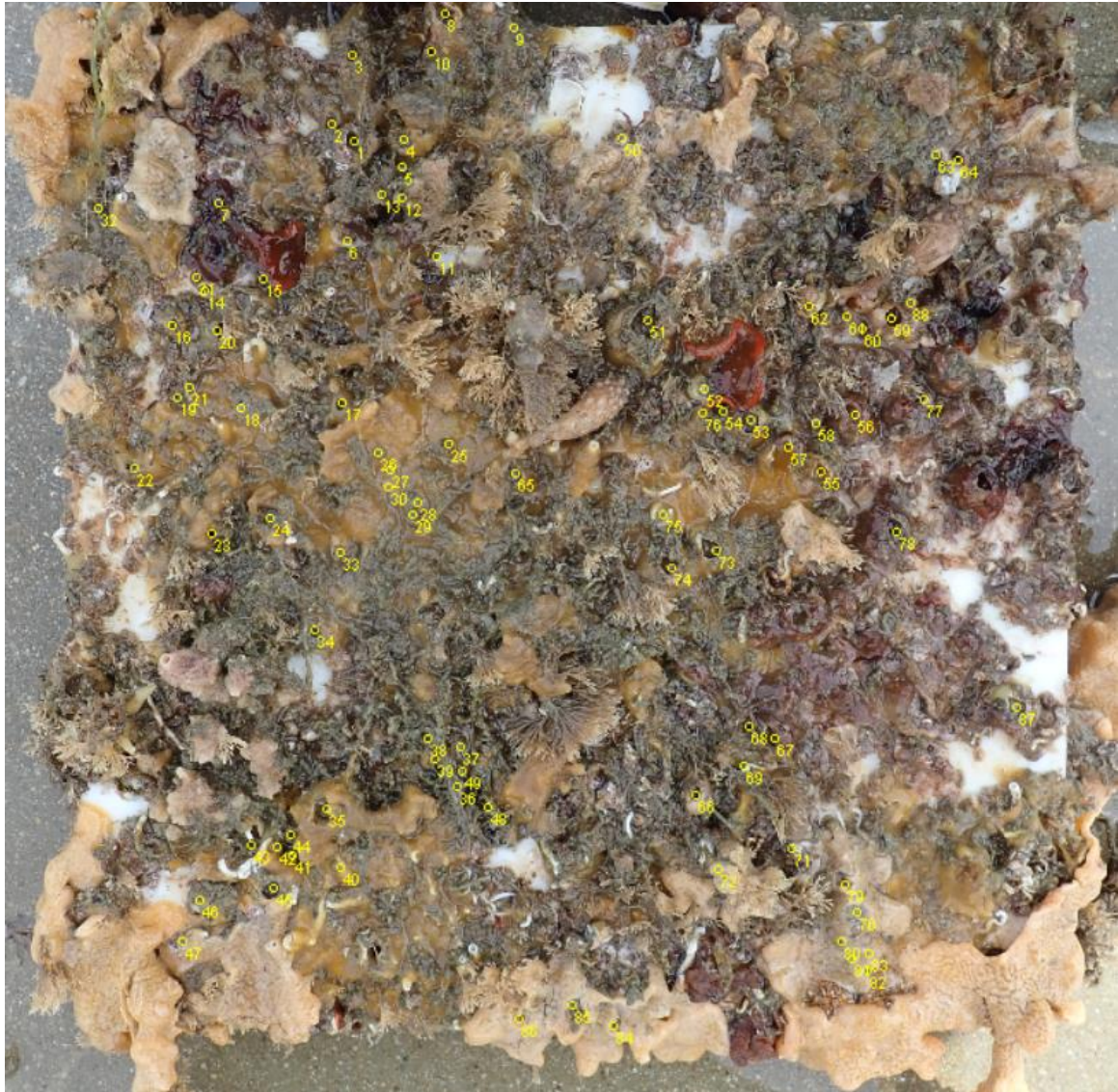

(4)

(5)

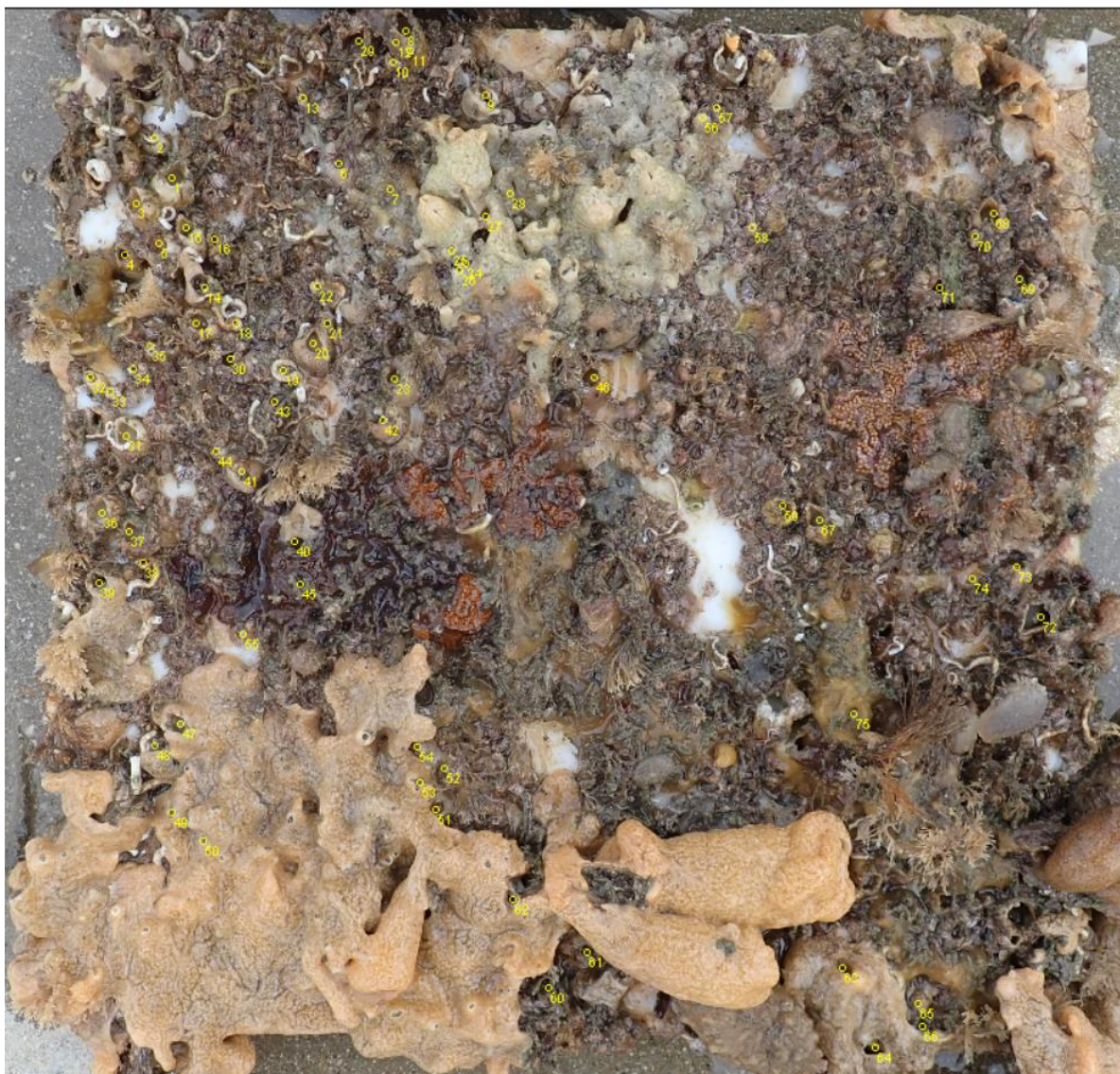

(6)

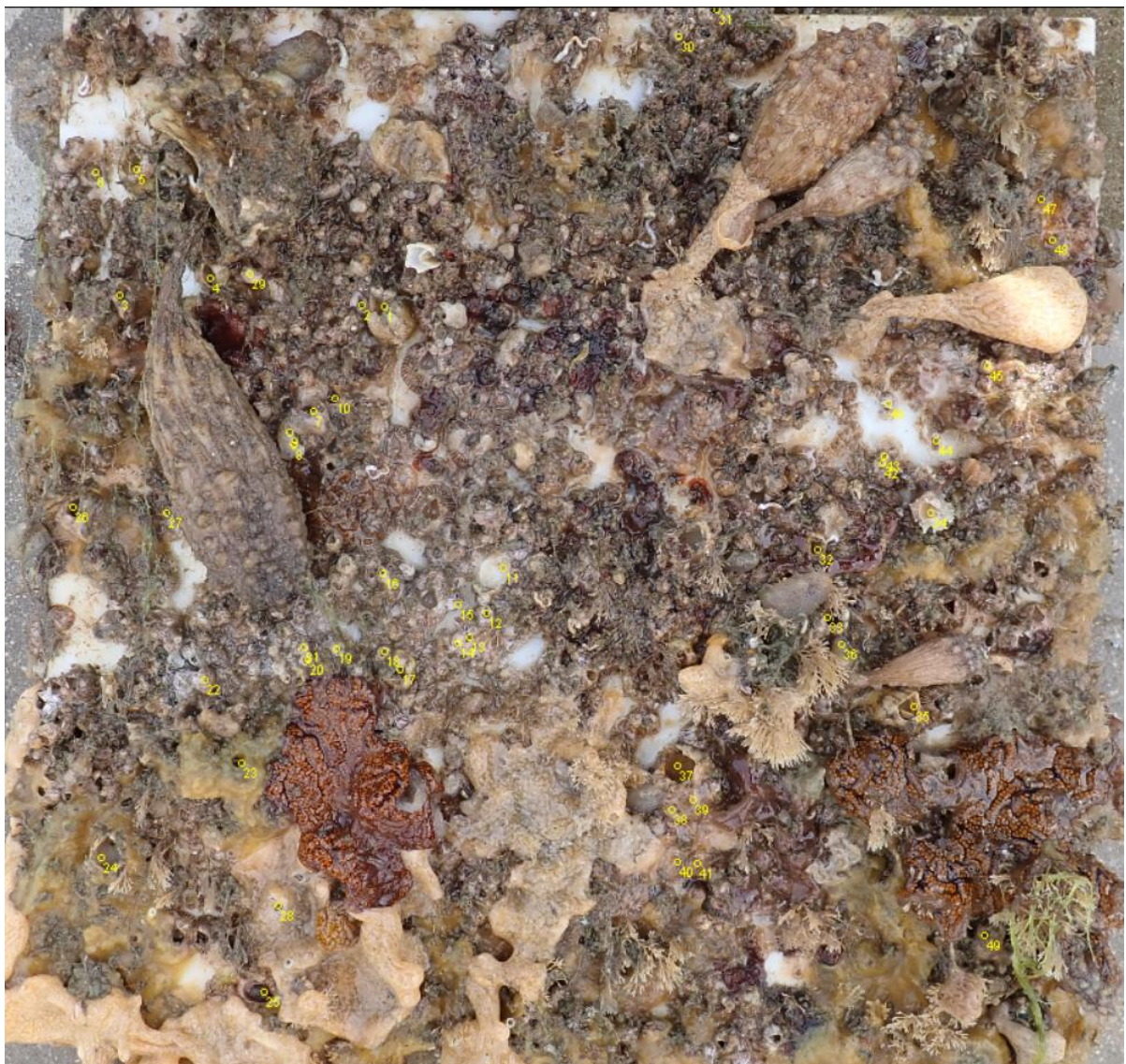

(7)

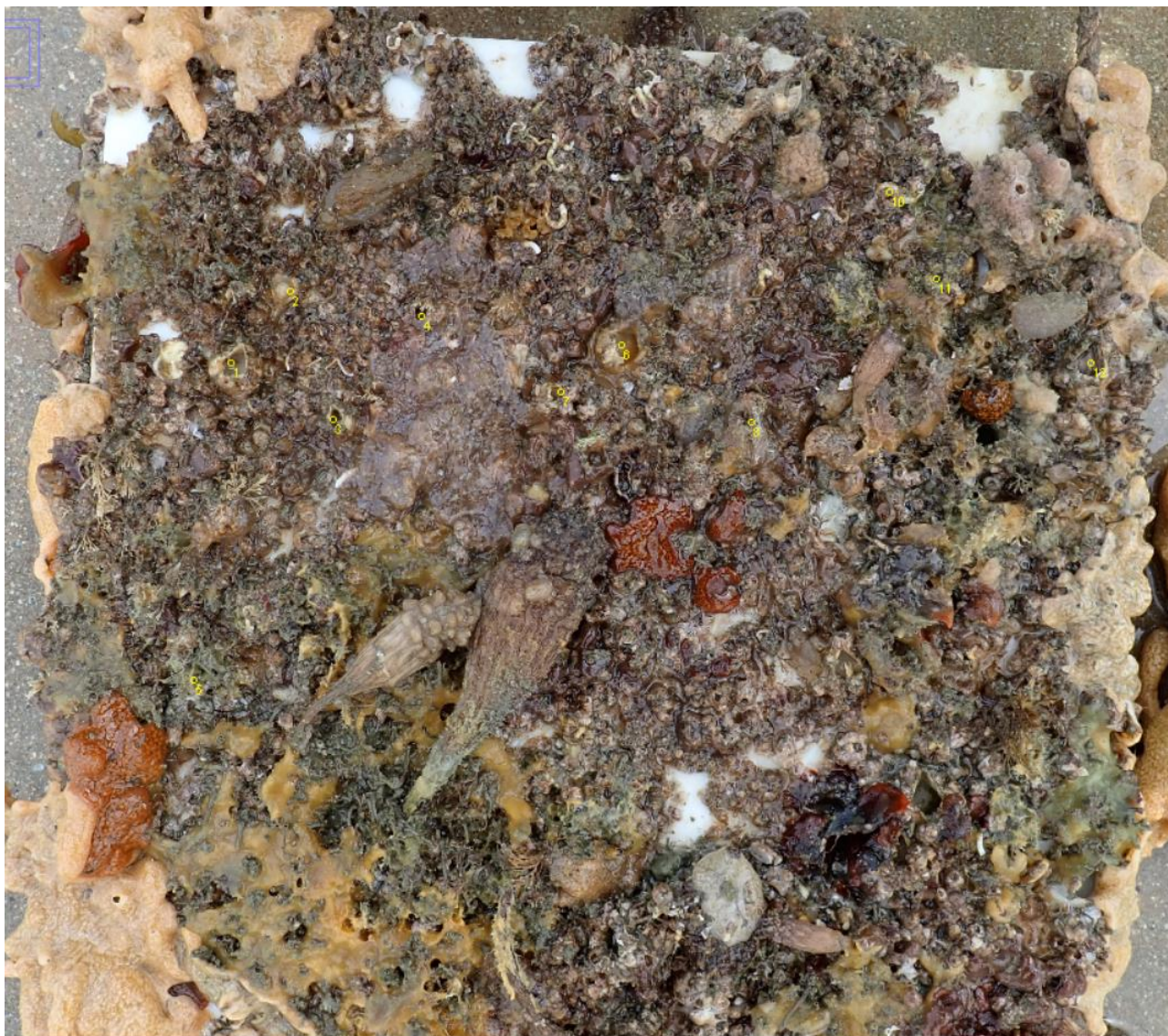

(8)

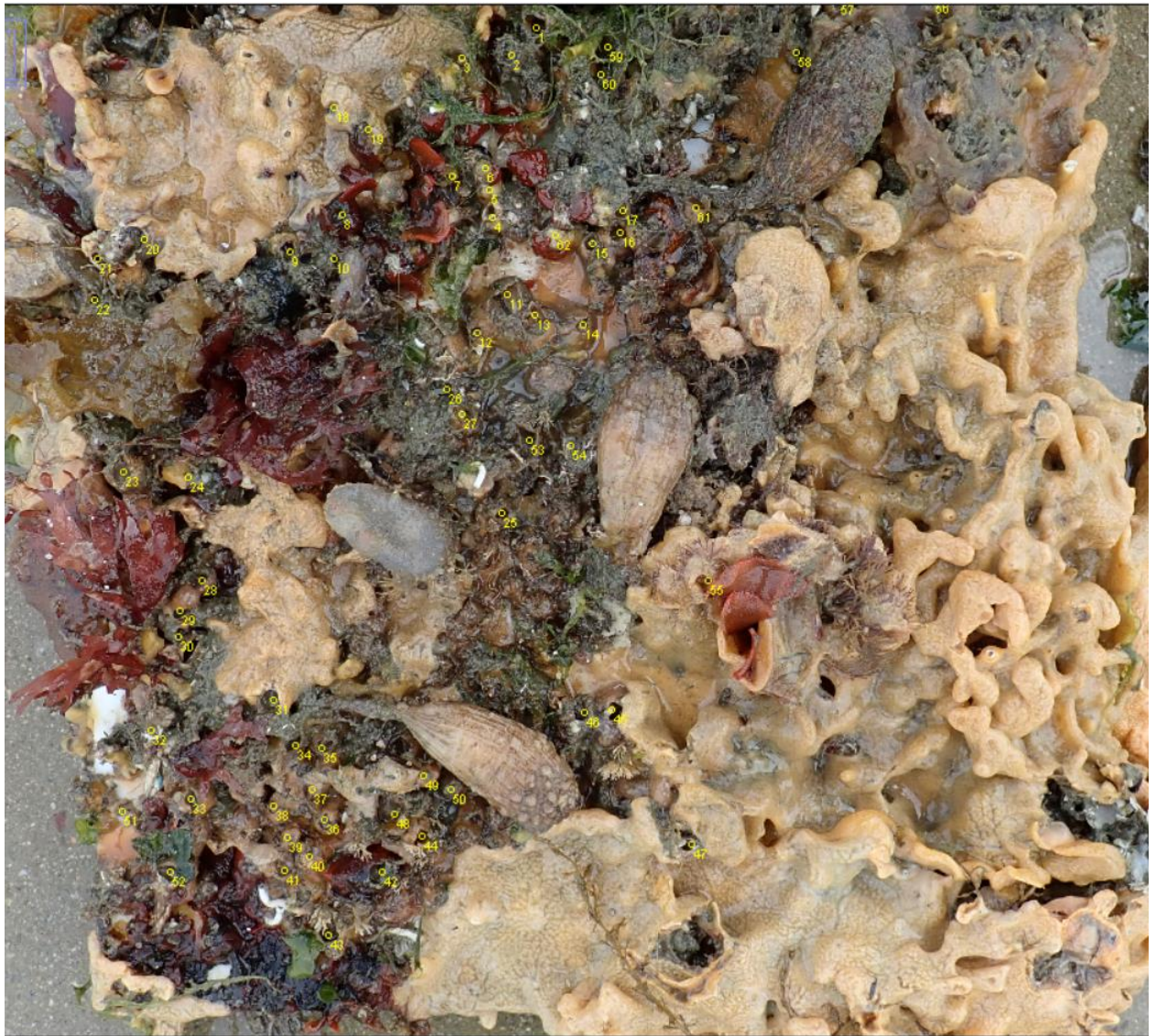

(9)

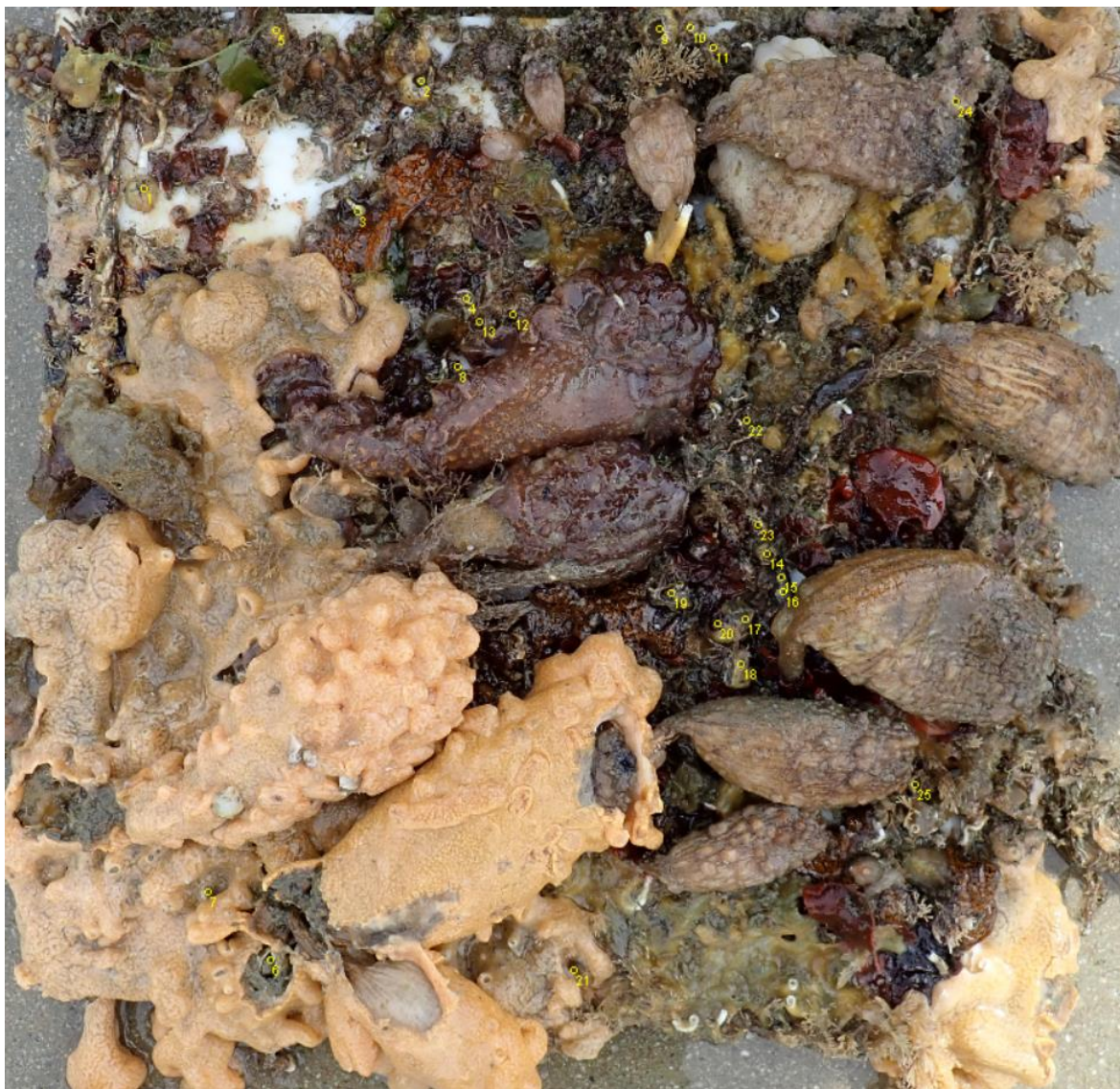

(10)

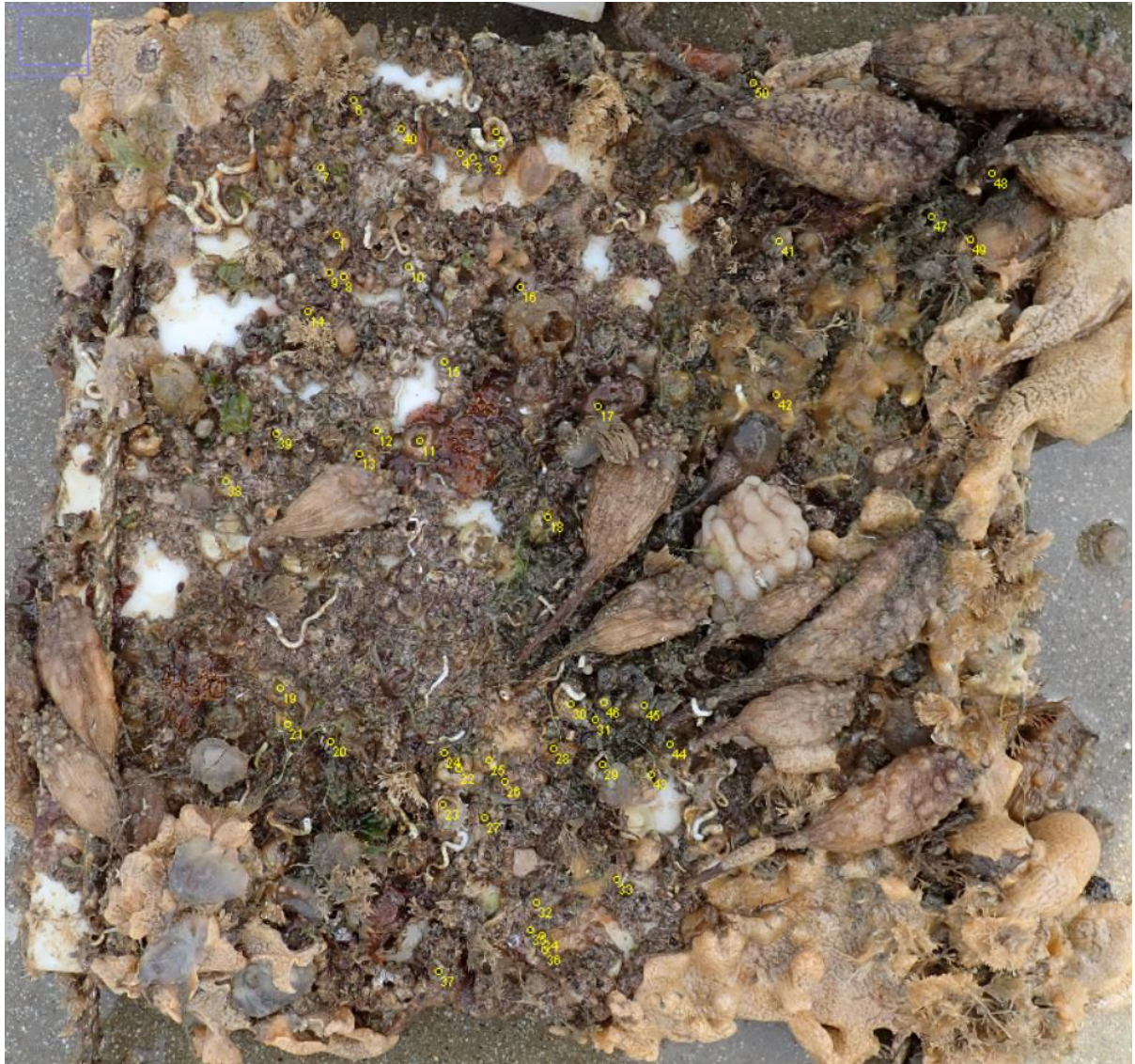

### 3. Sokcho

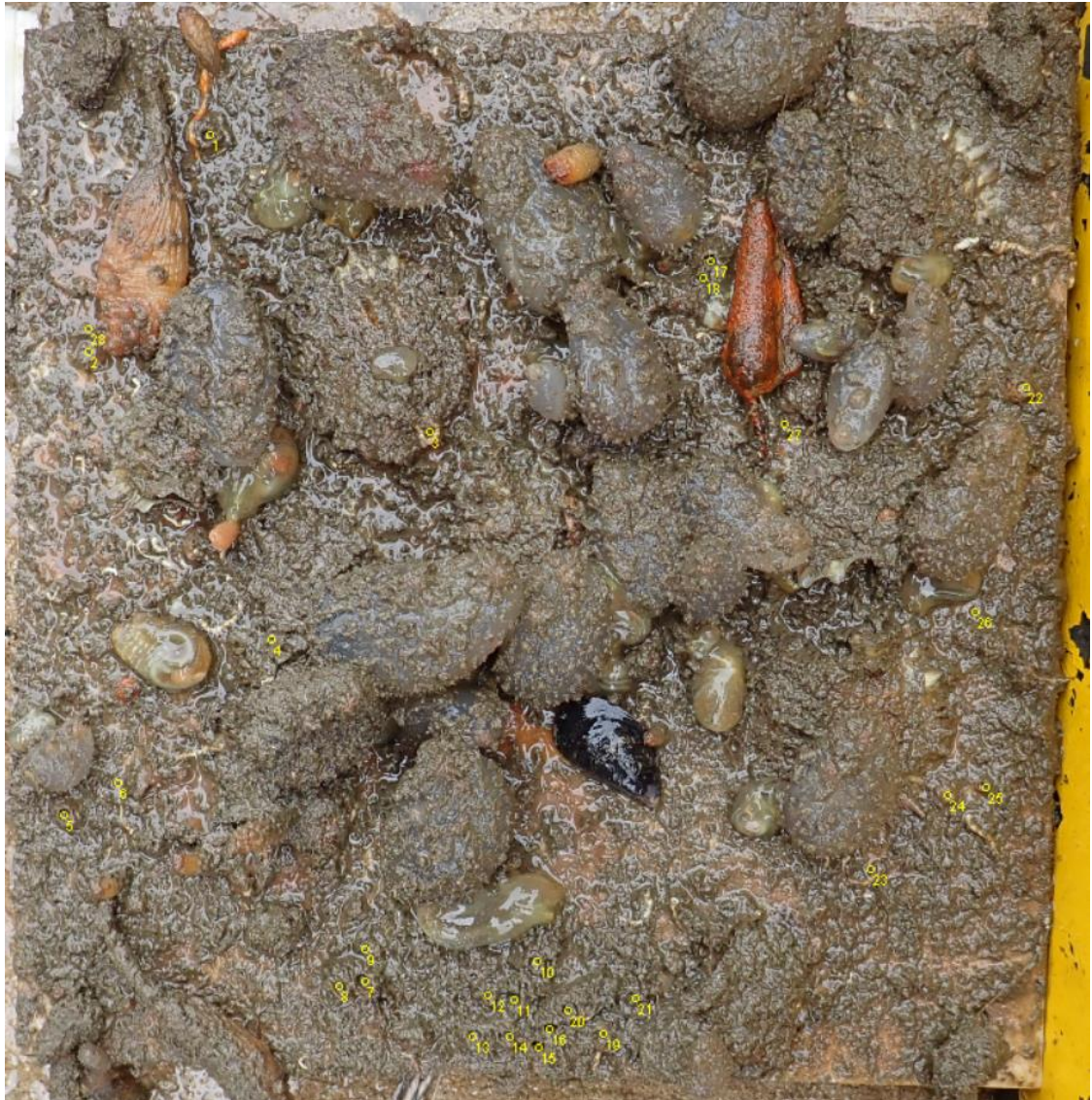

(1)

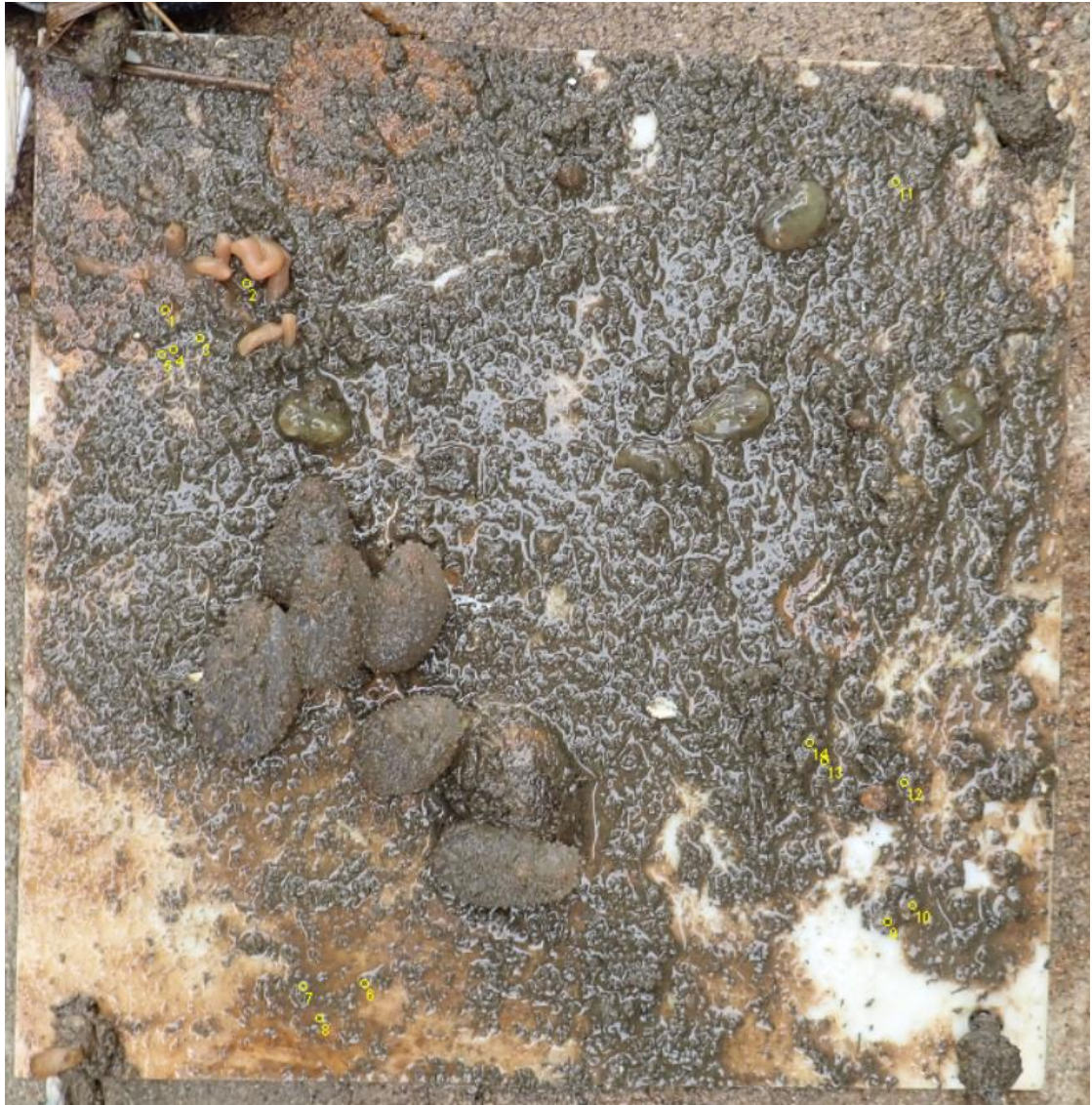

(2)

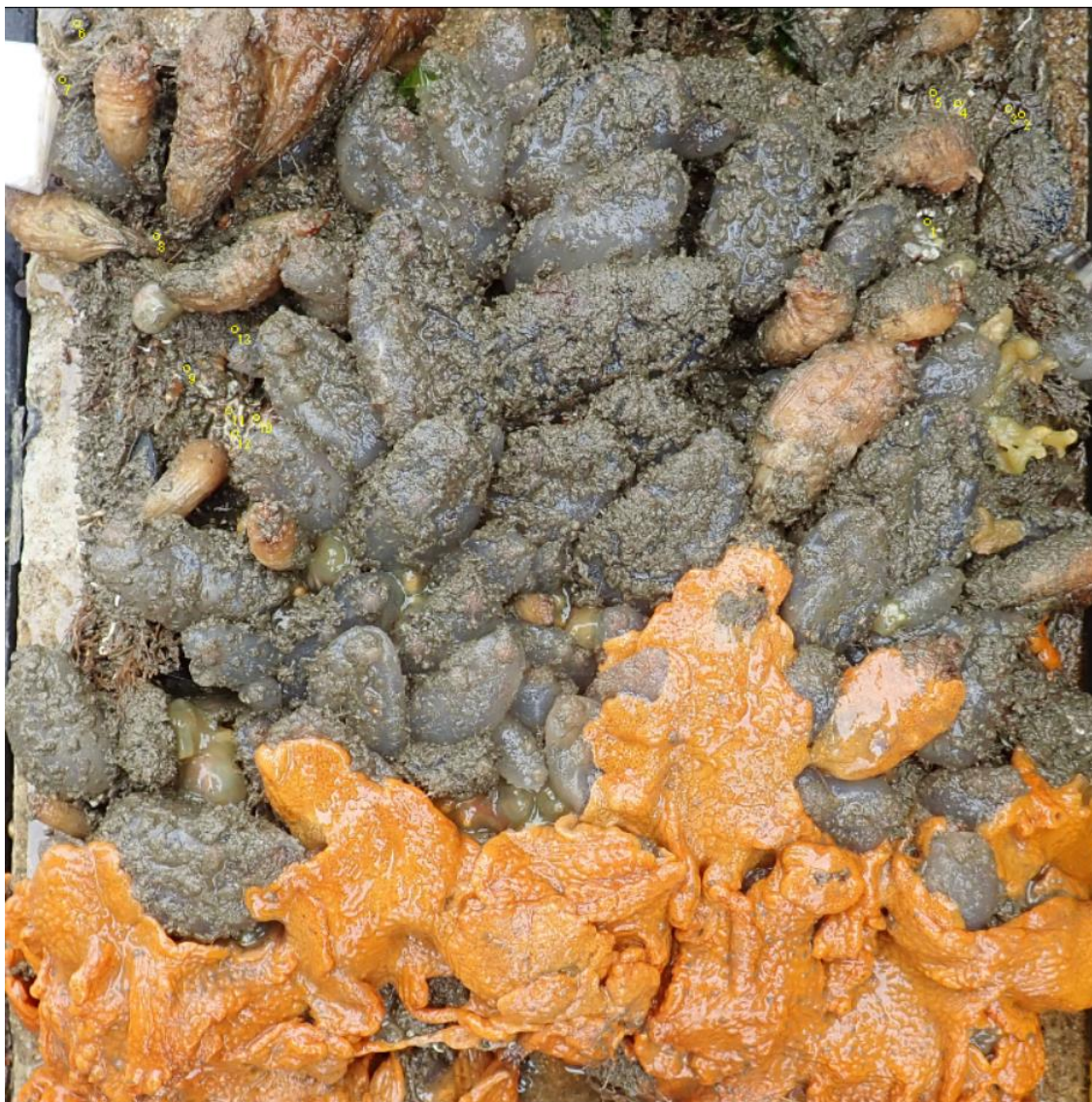

(3)

(4)

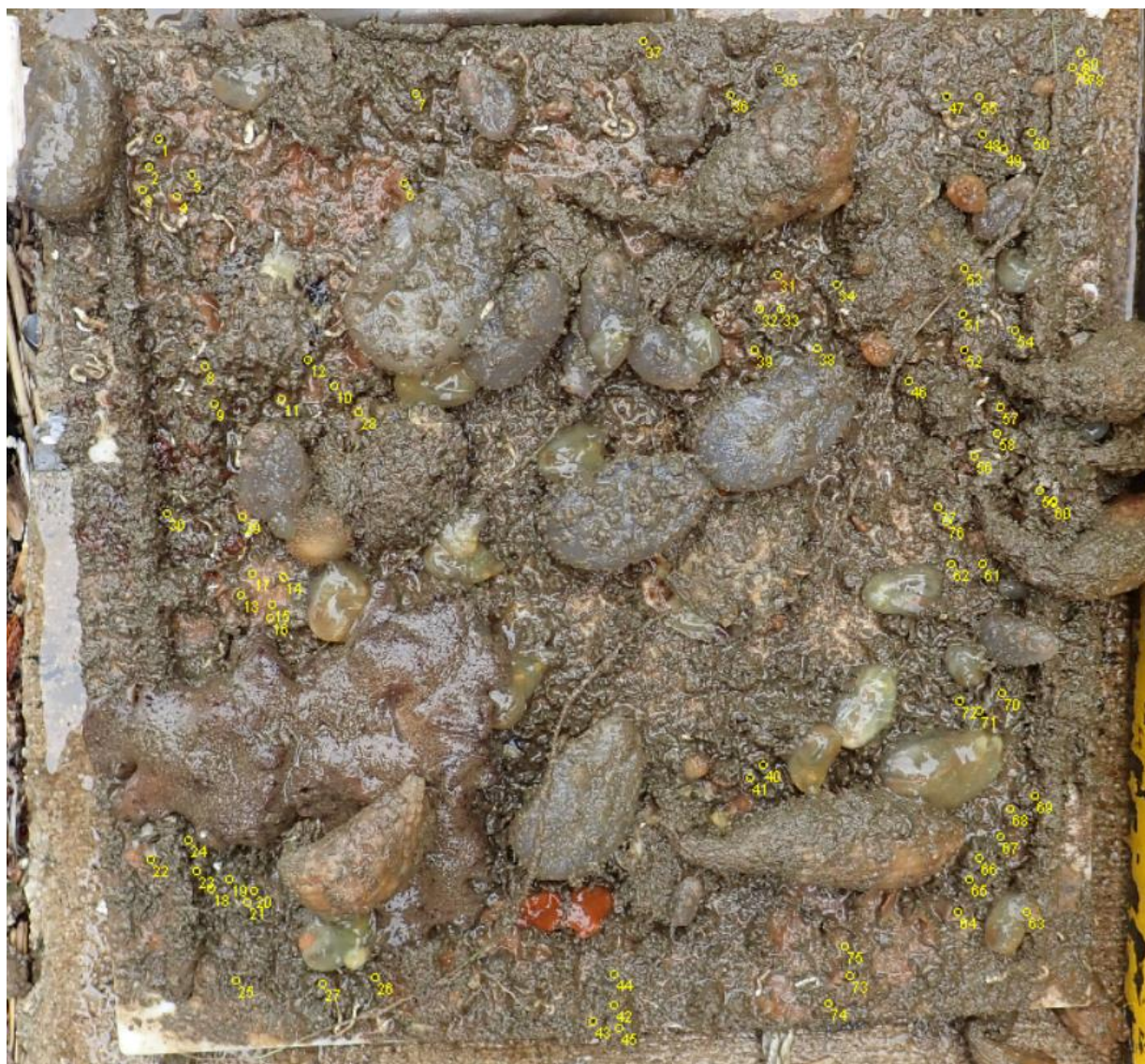

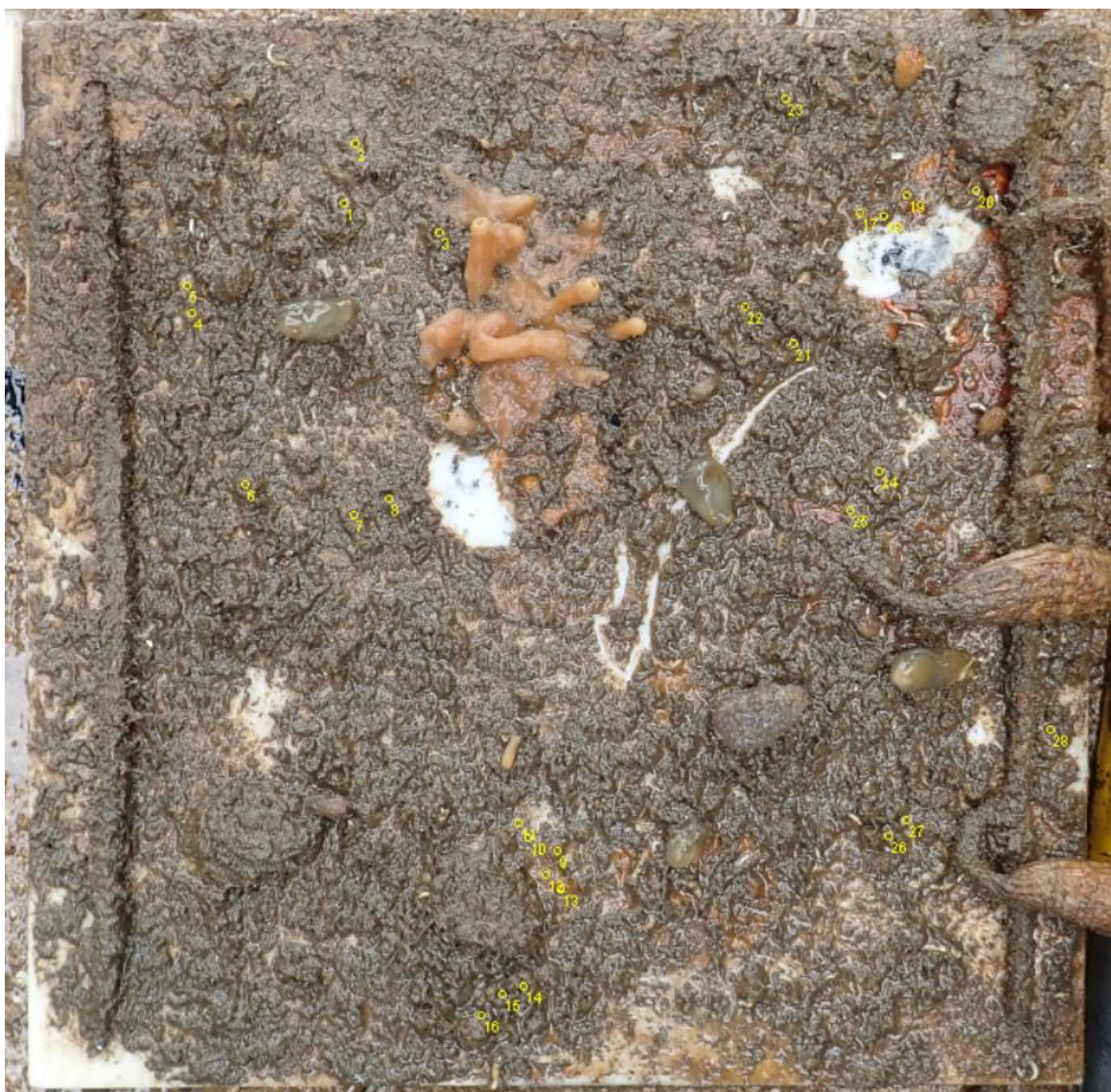

(5)

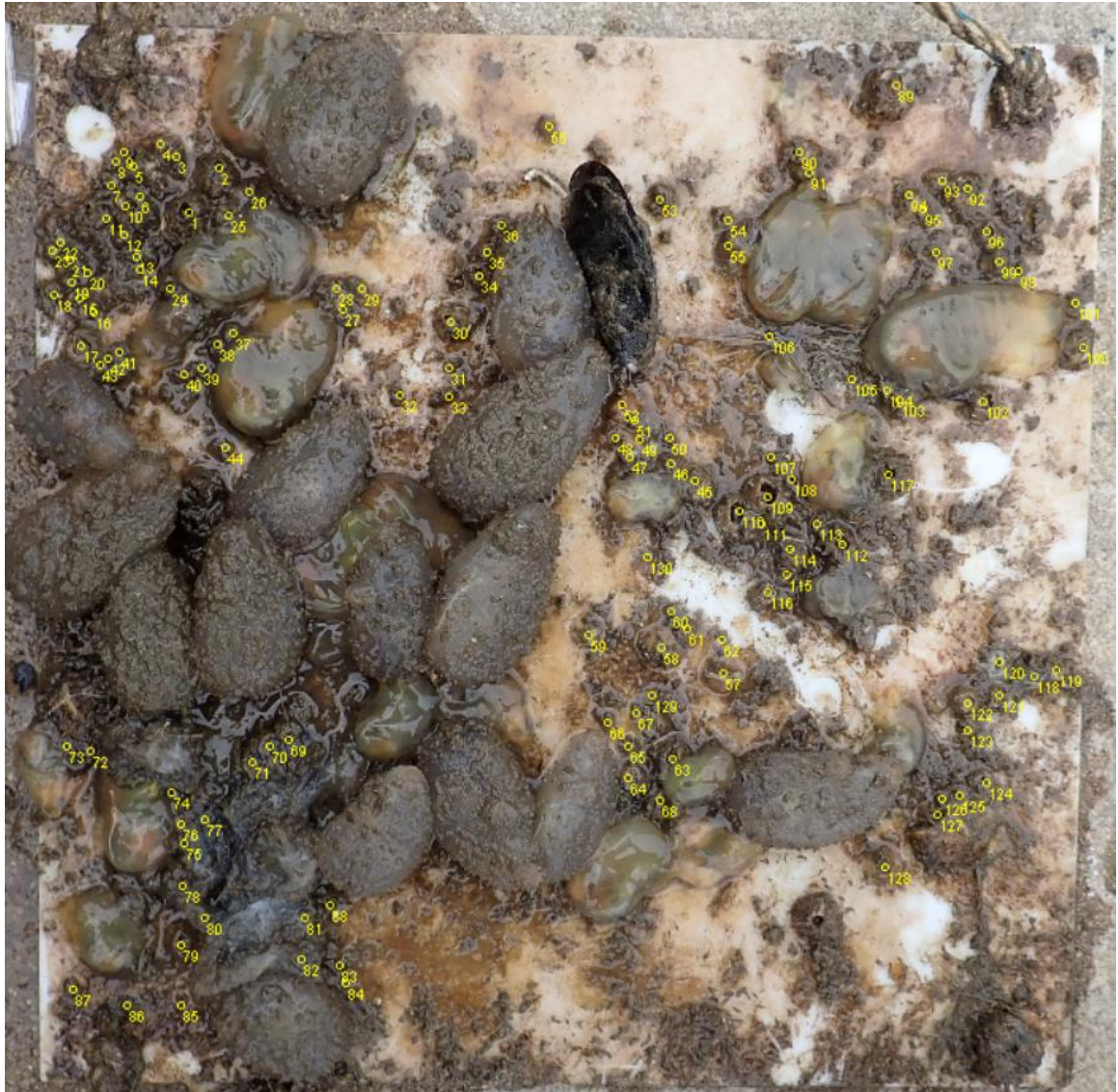

(6)

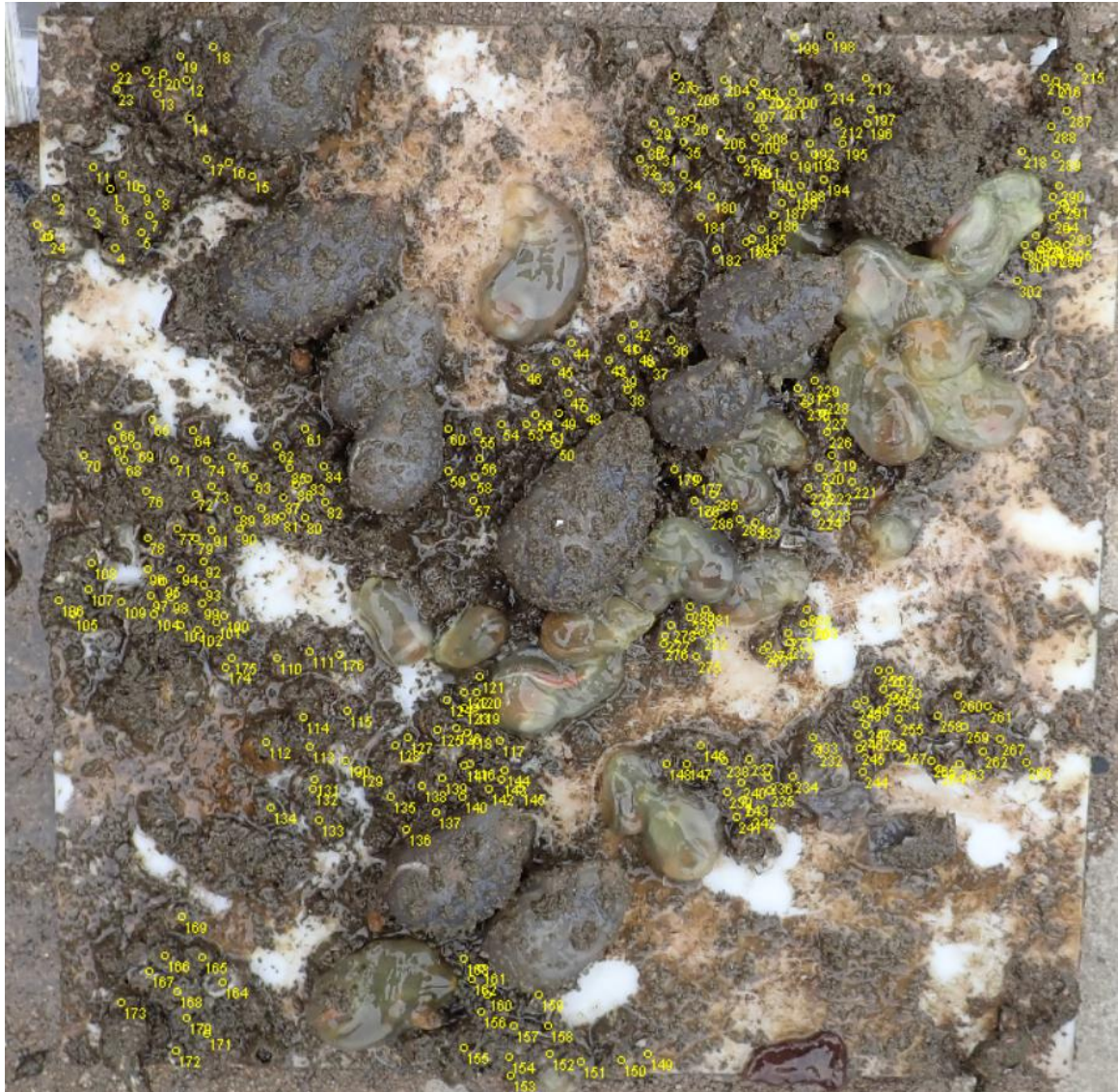

(7)

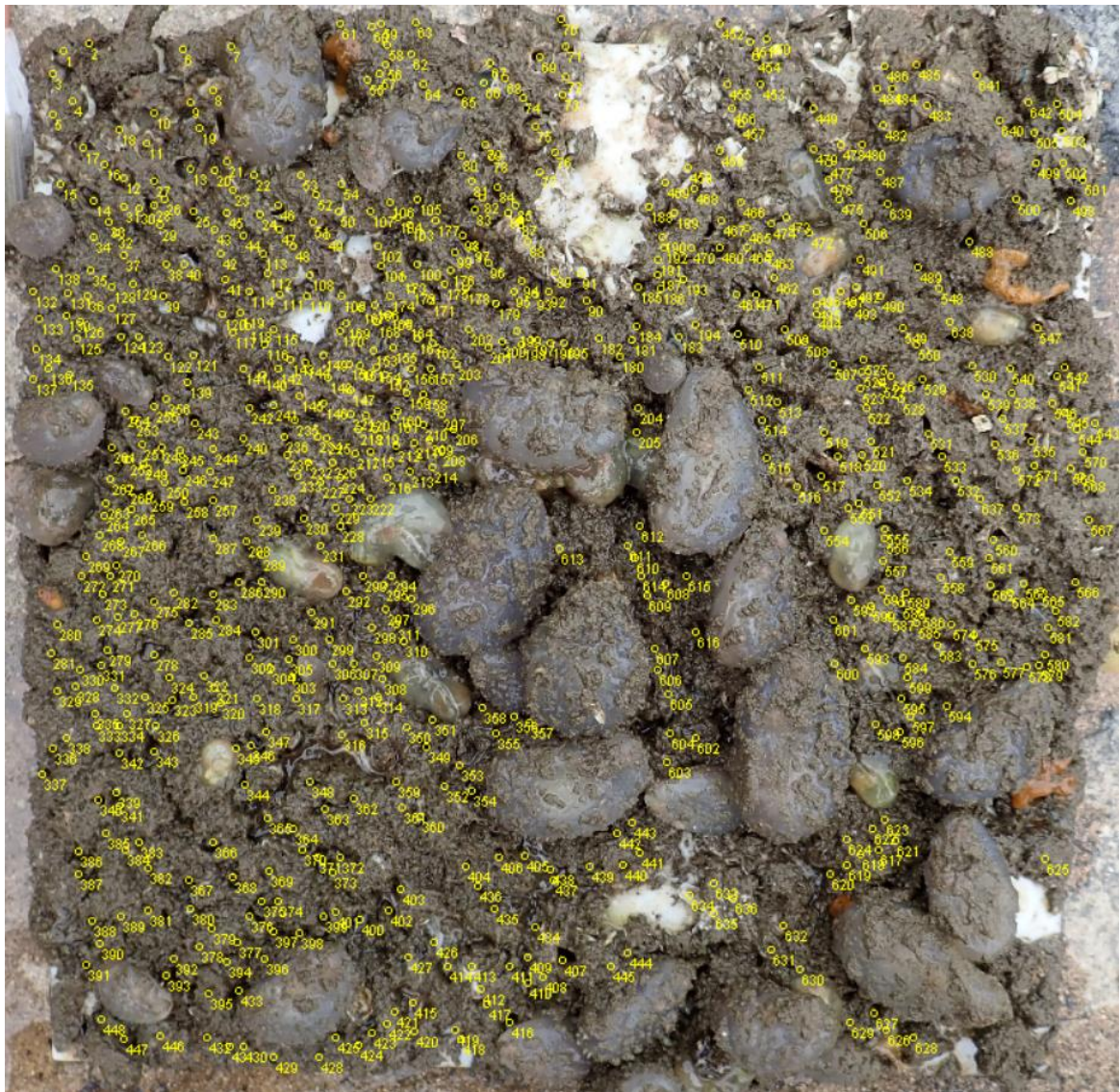

(8)

(9)

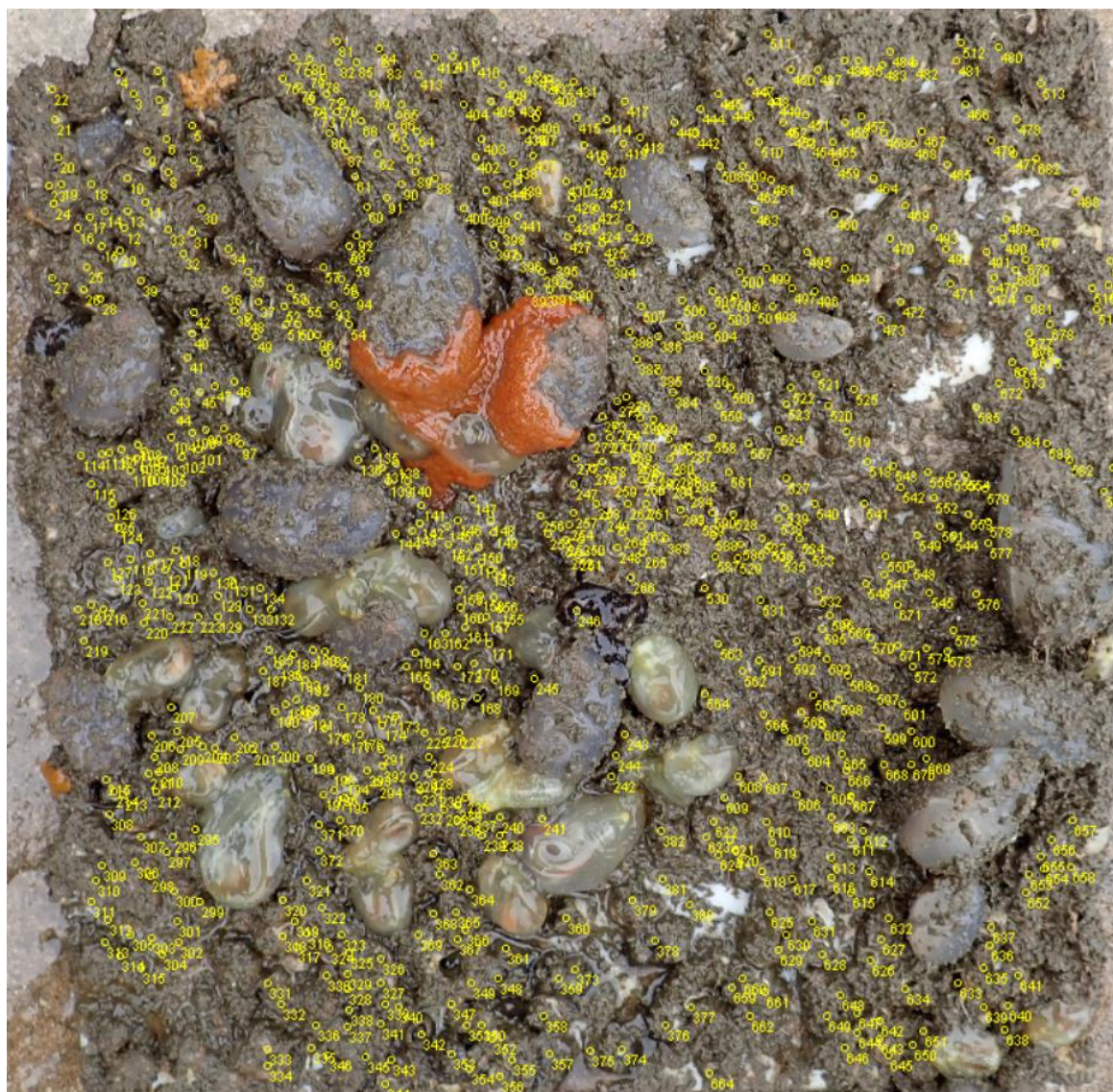

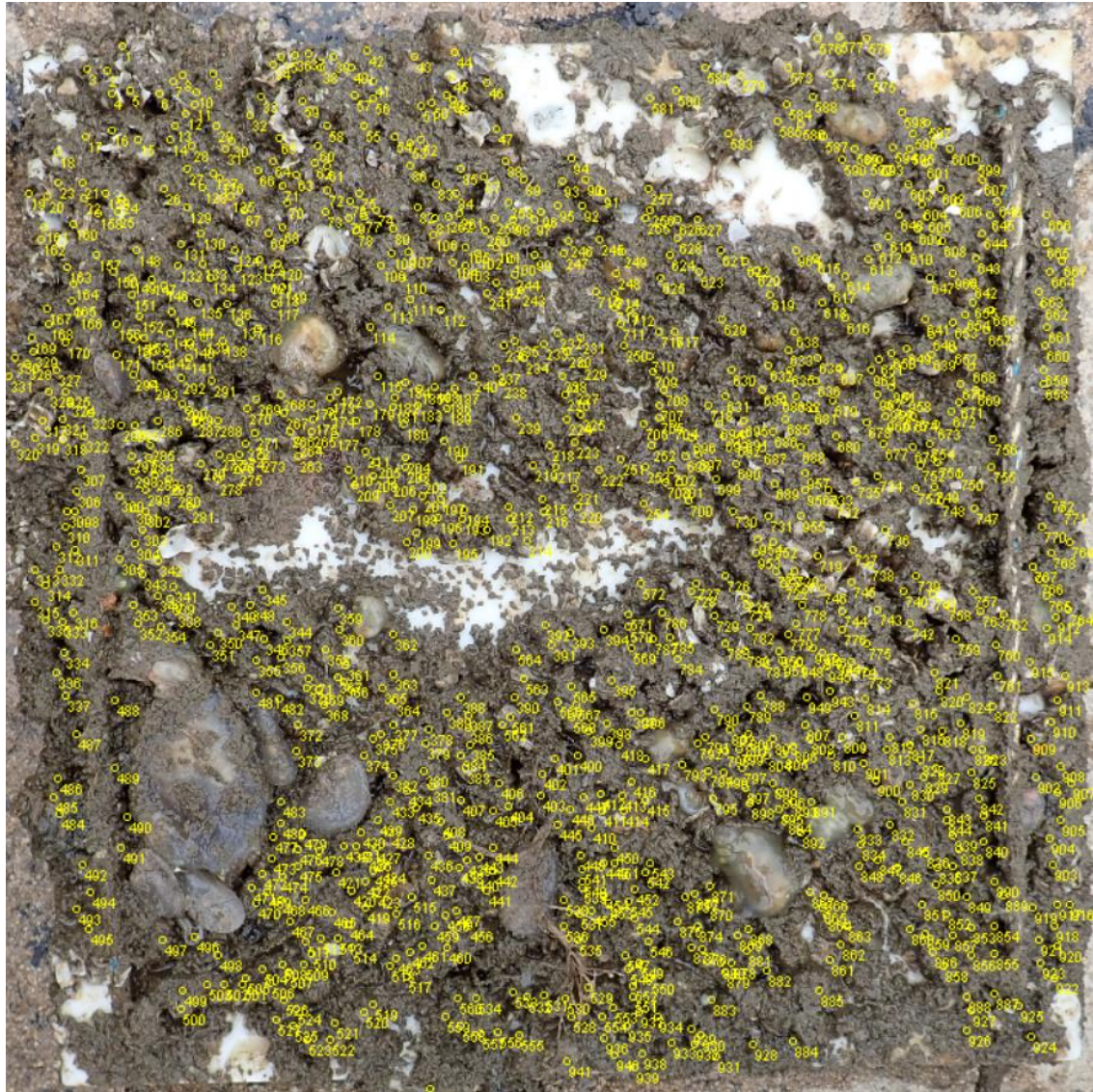

(10)

## 4. Hanlim

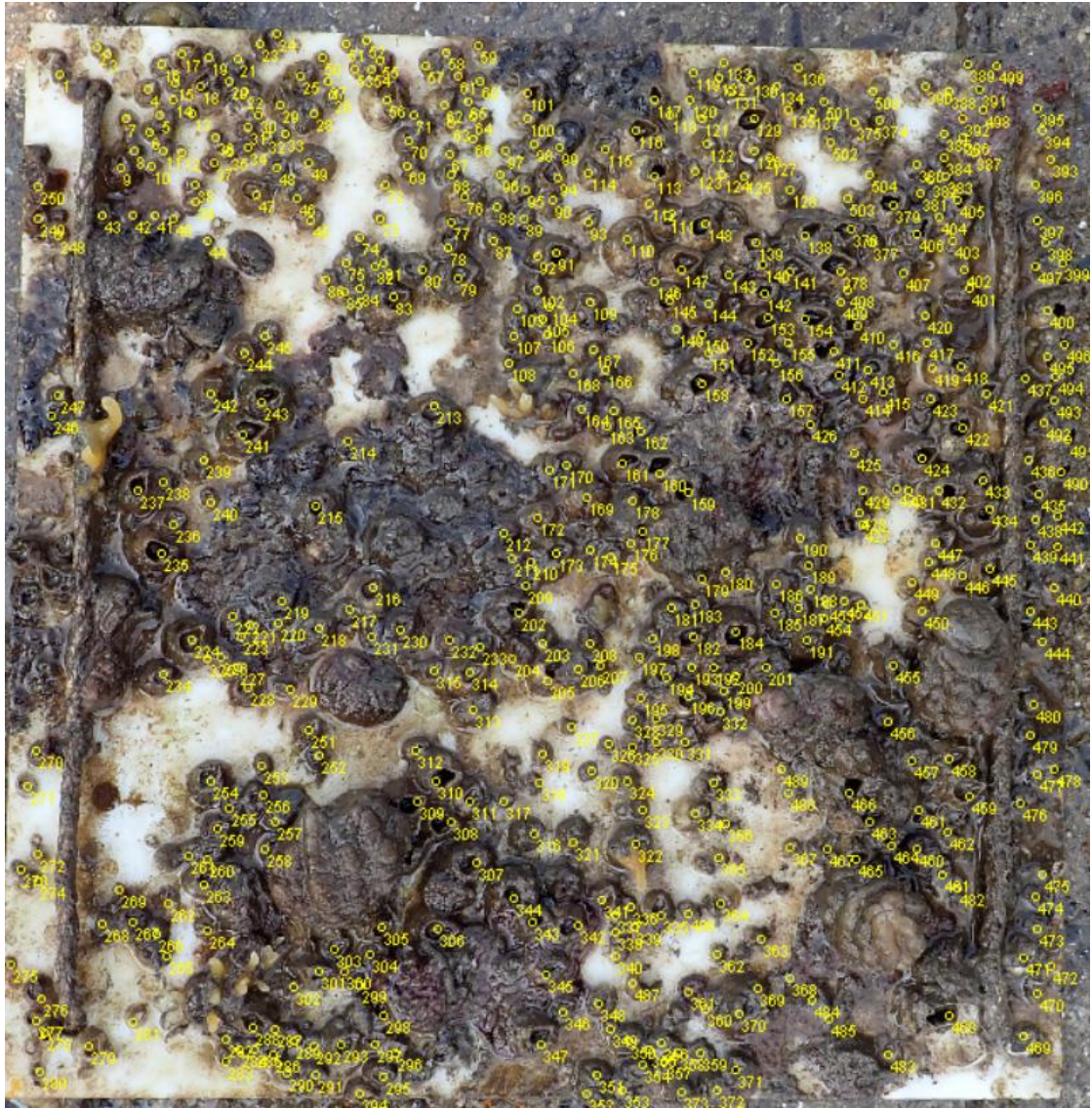

(1)

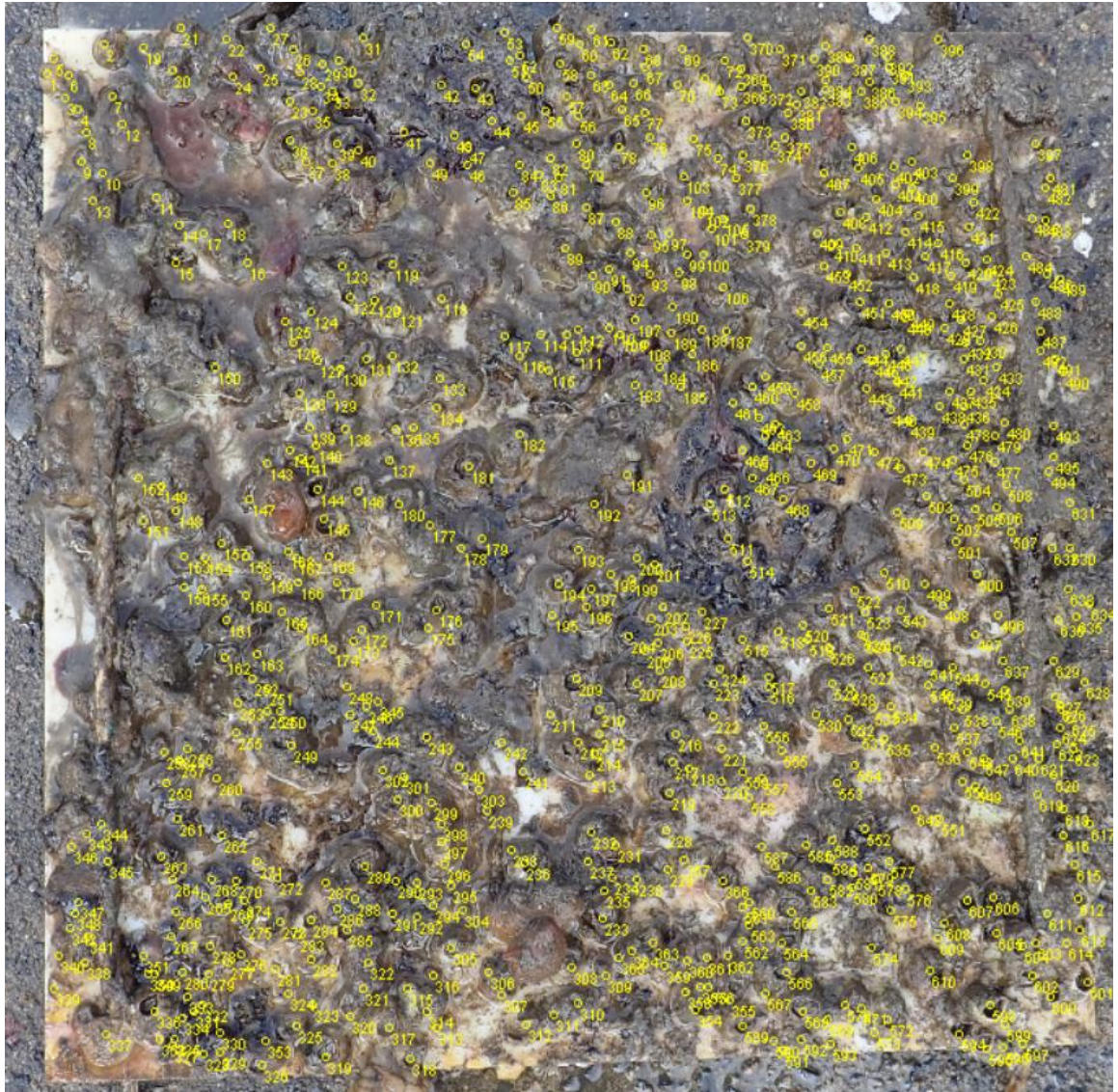

(2)

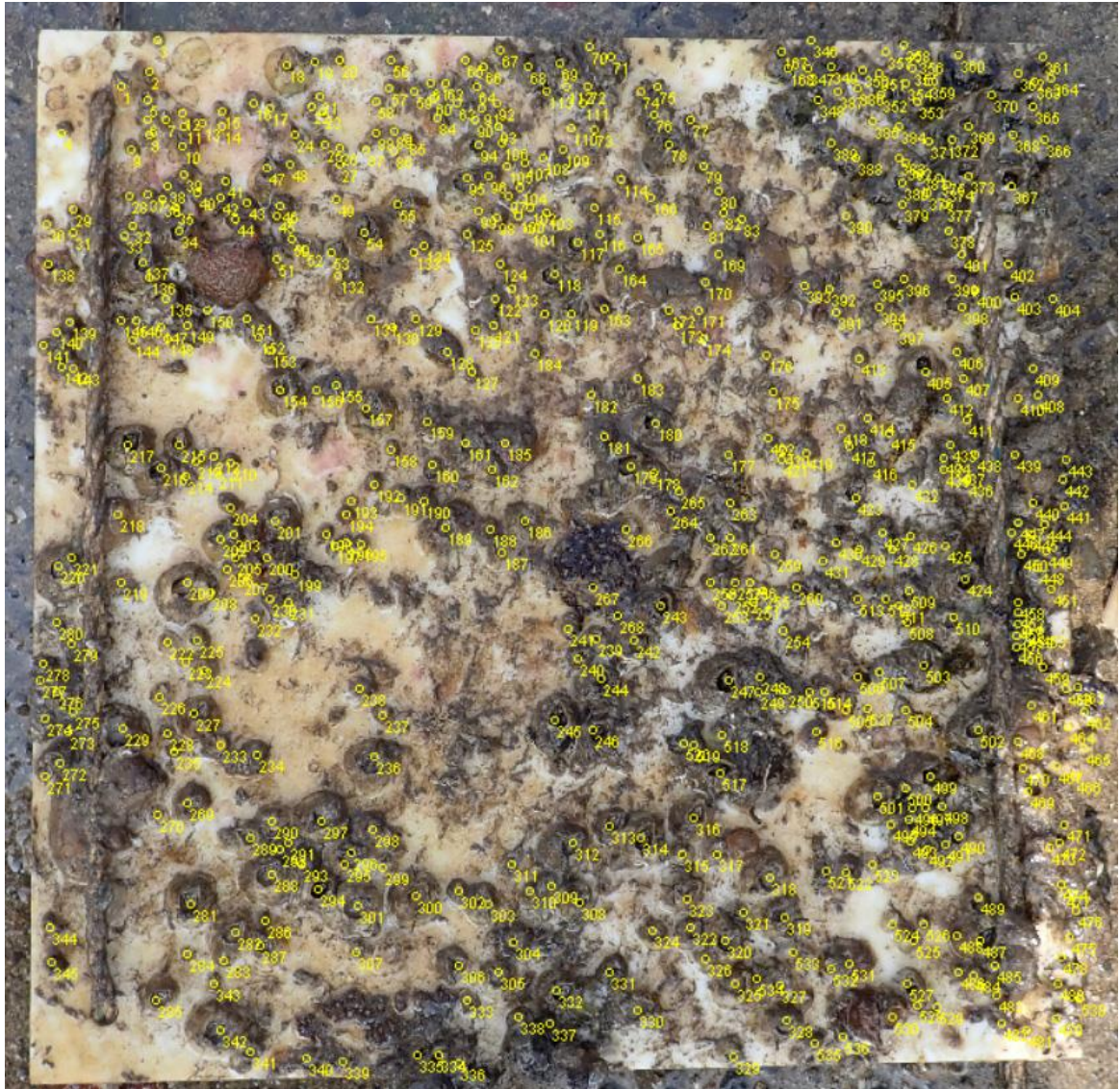

(3)

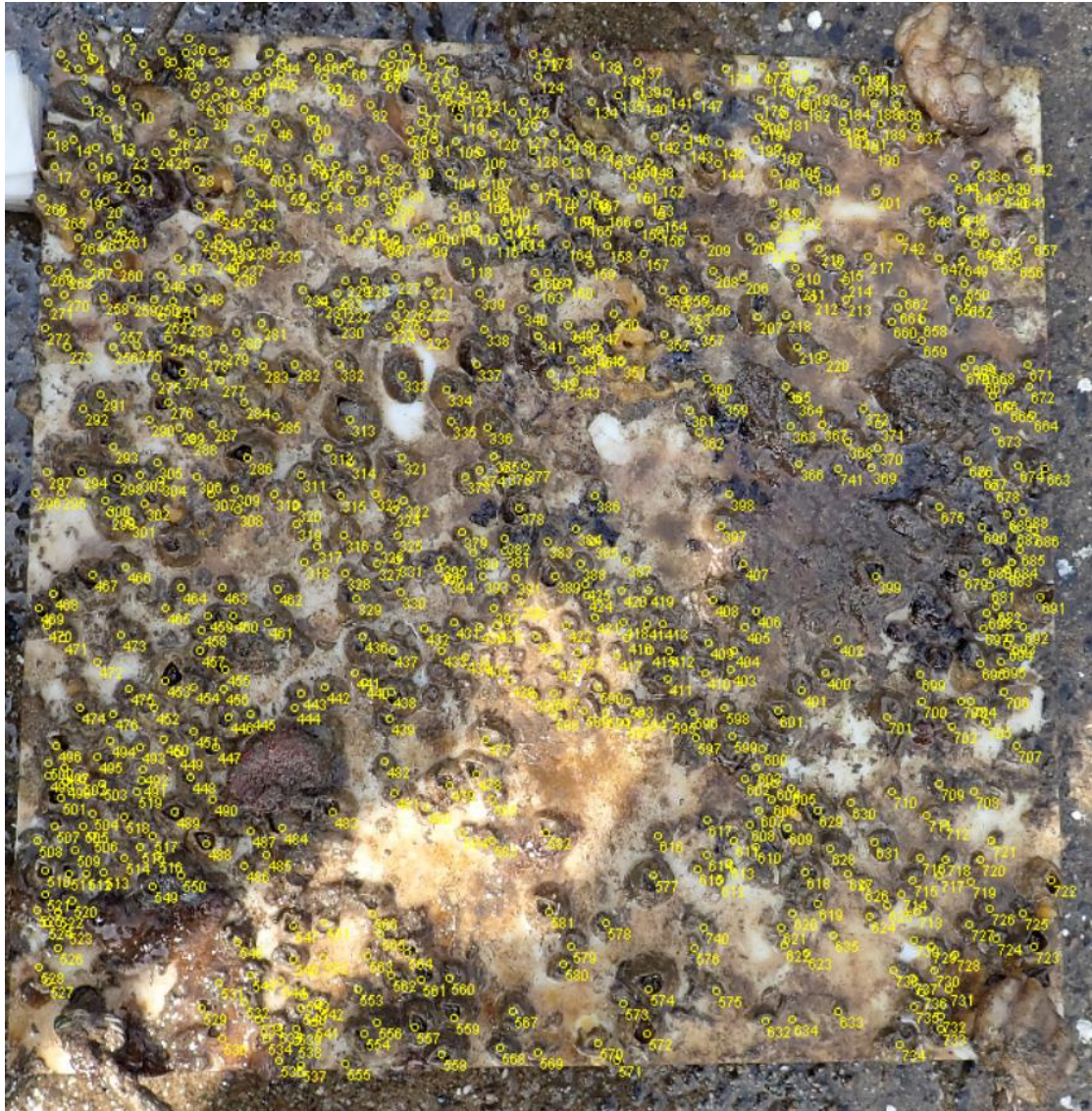

(4)

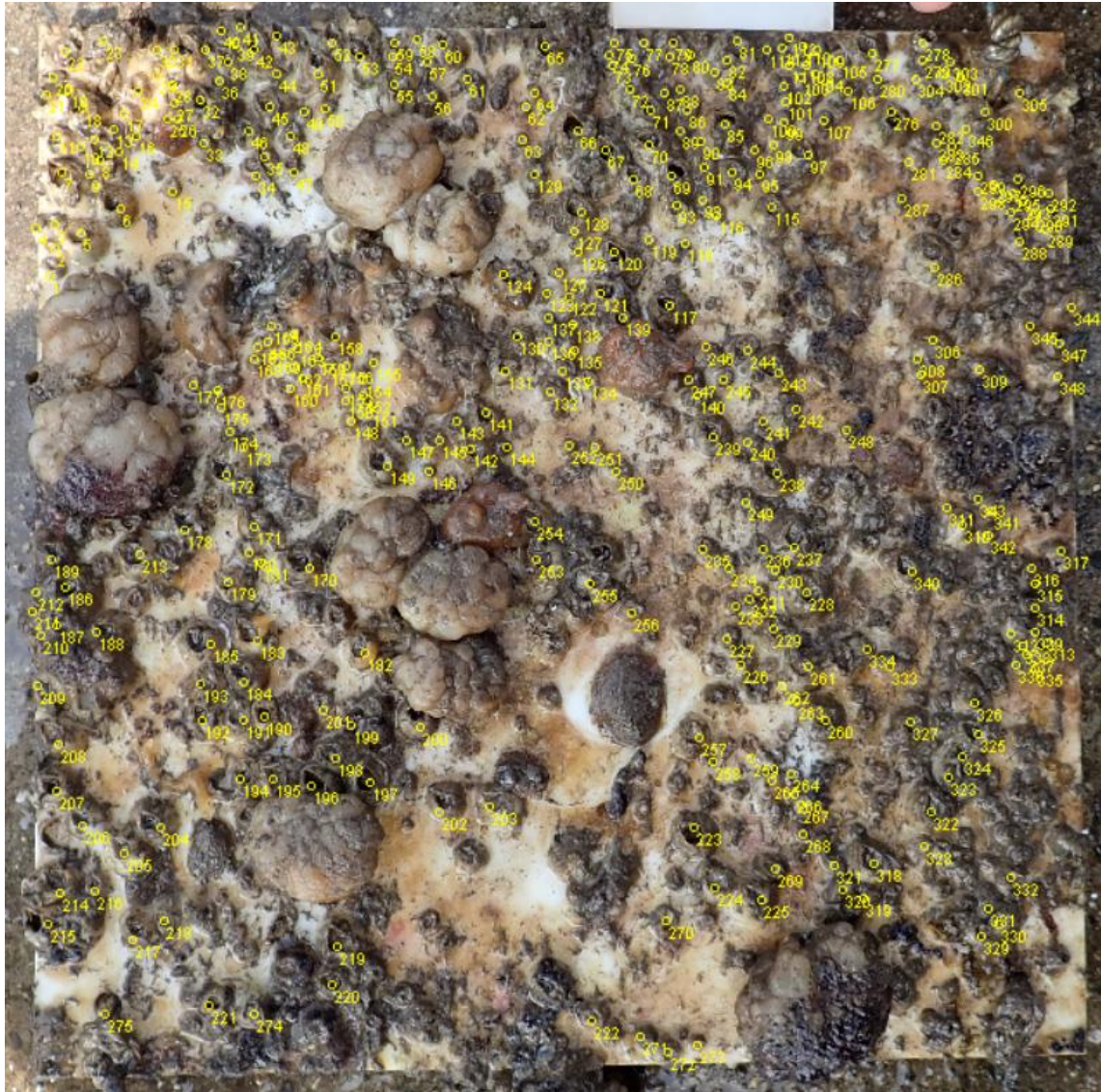

(5)

(6)

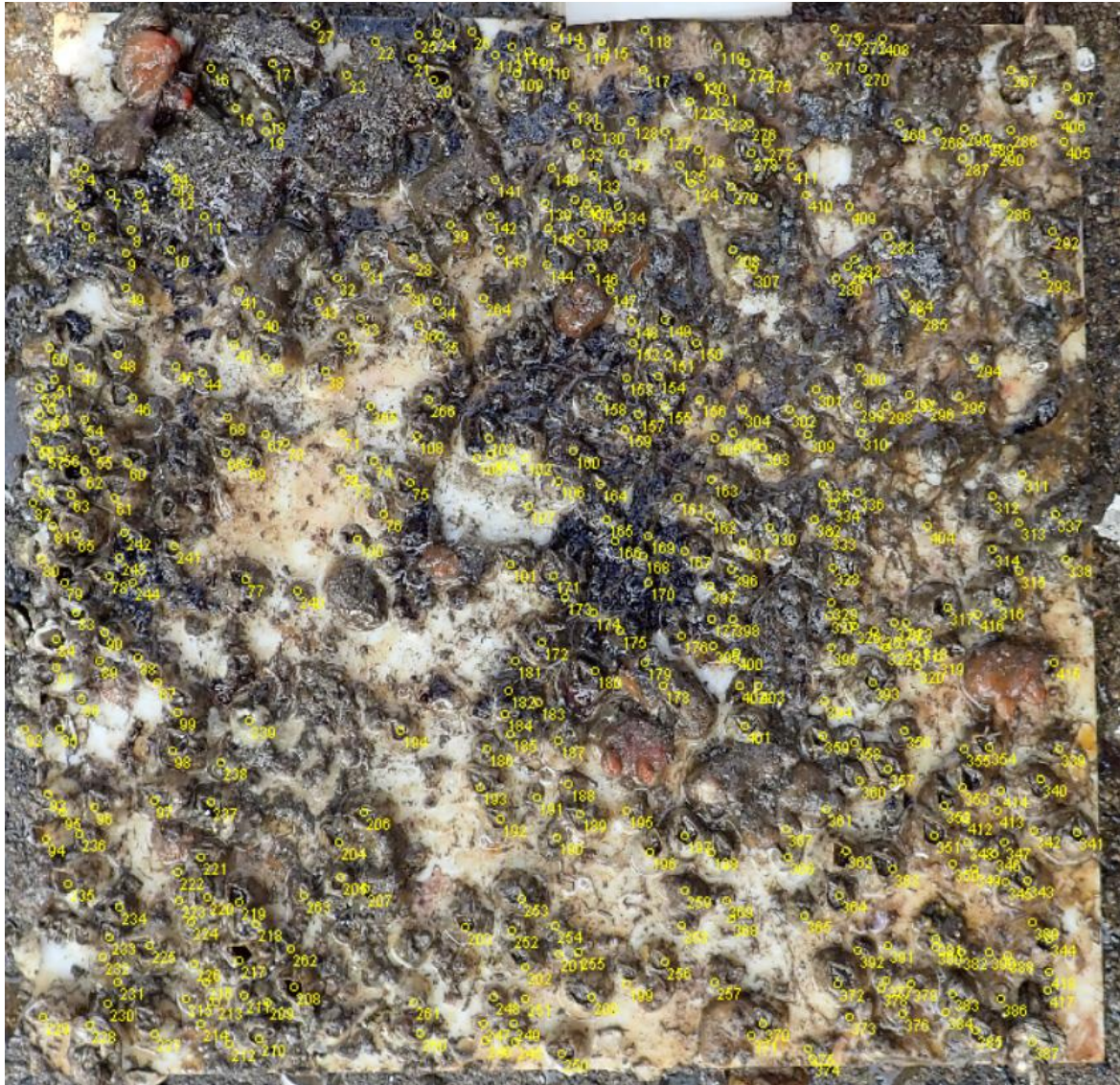

(7)

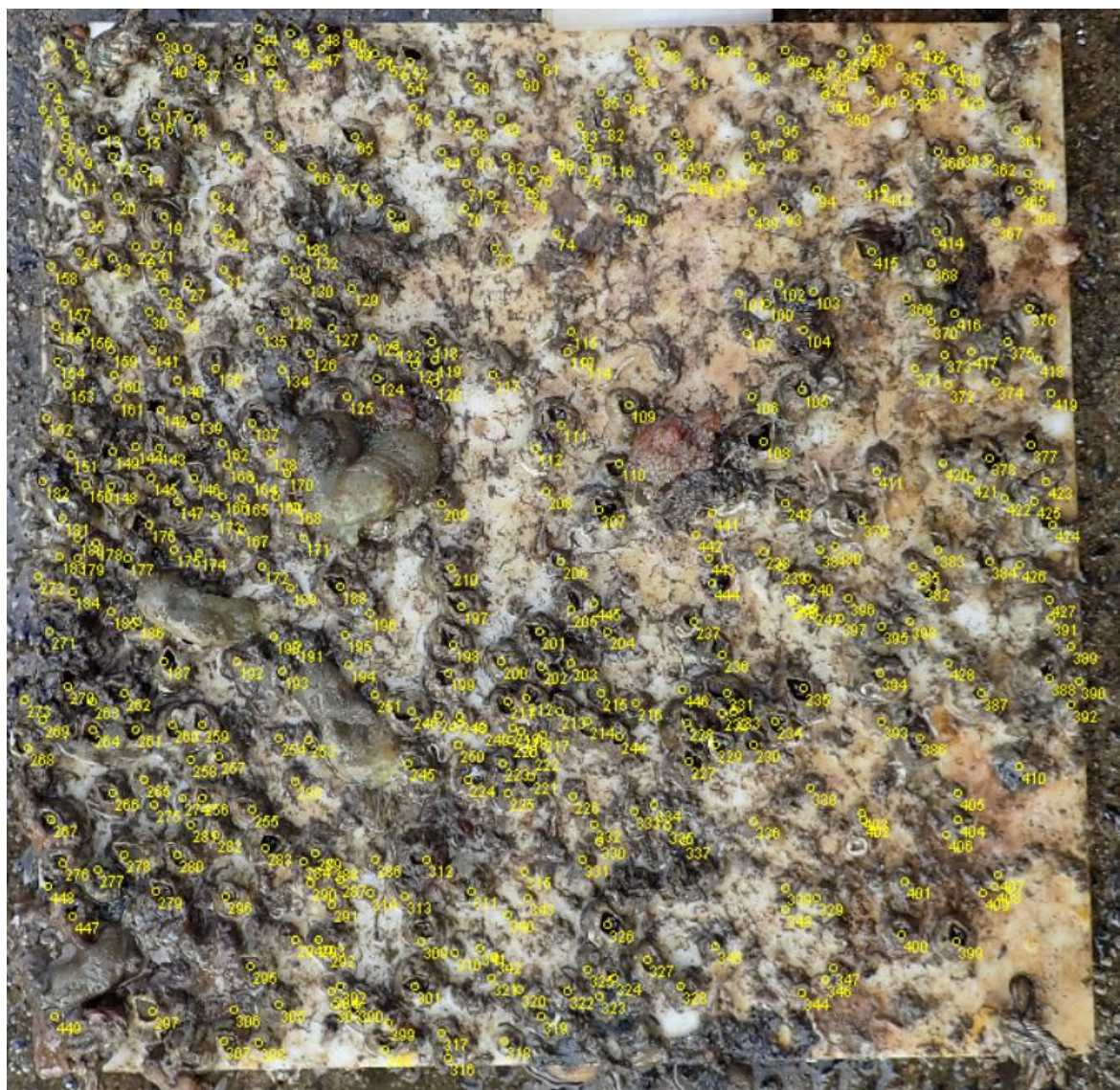

(8)

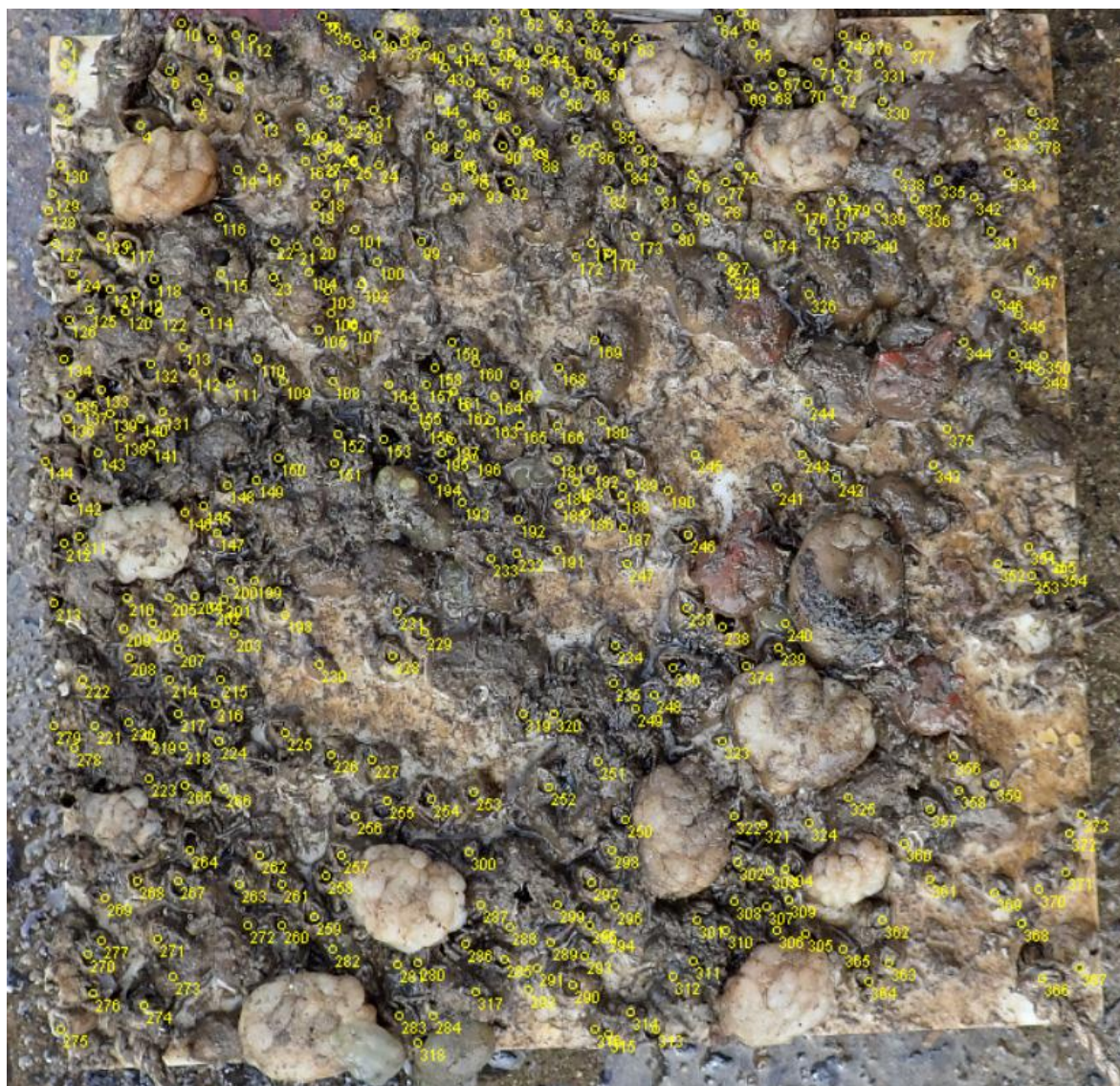

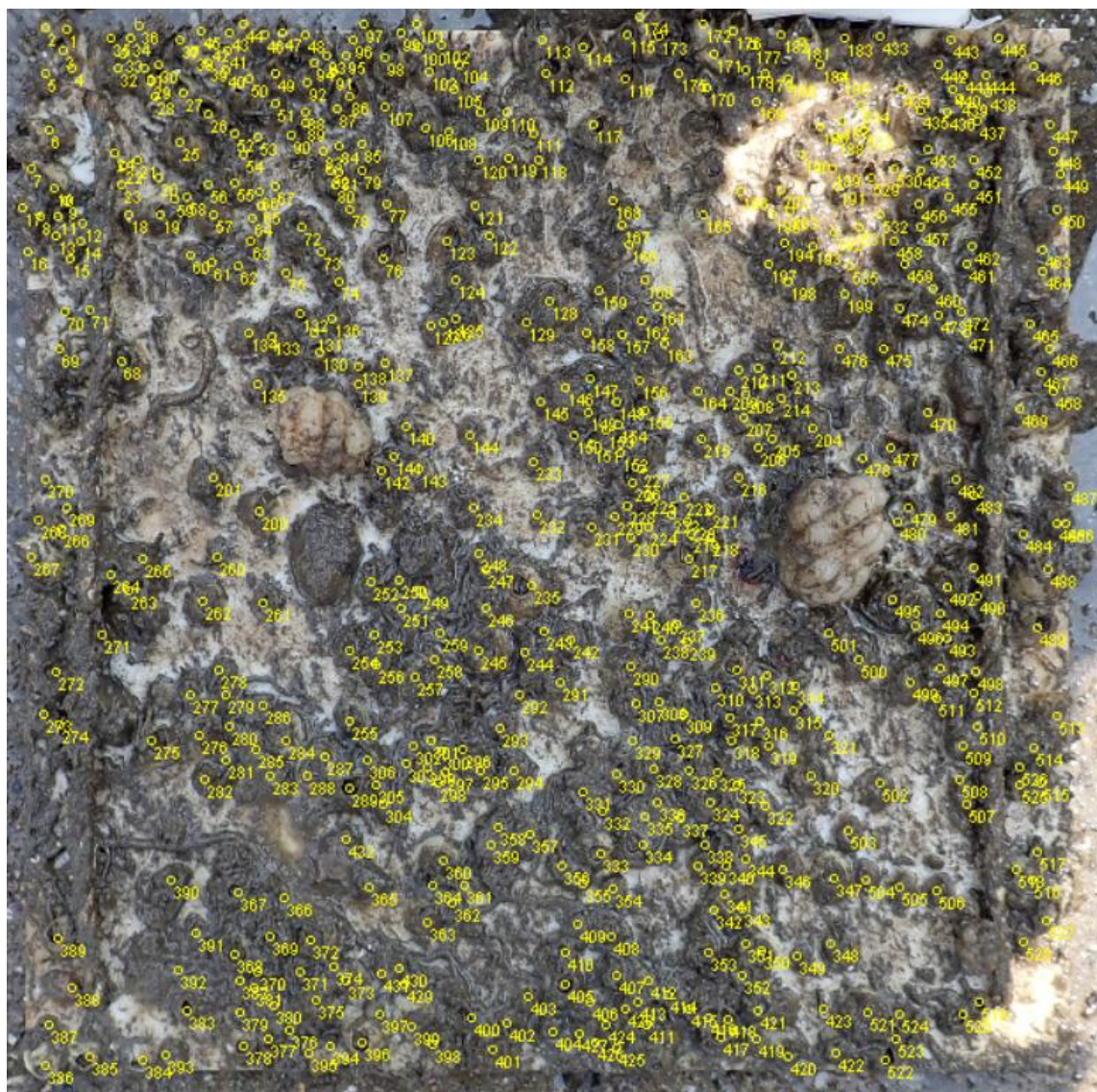

(9)

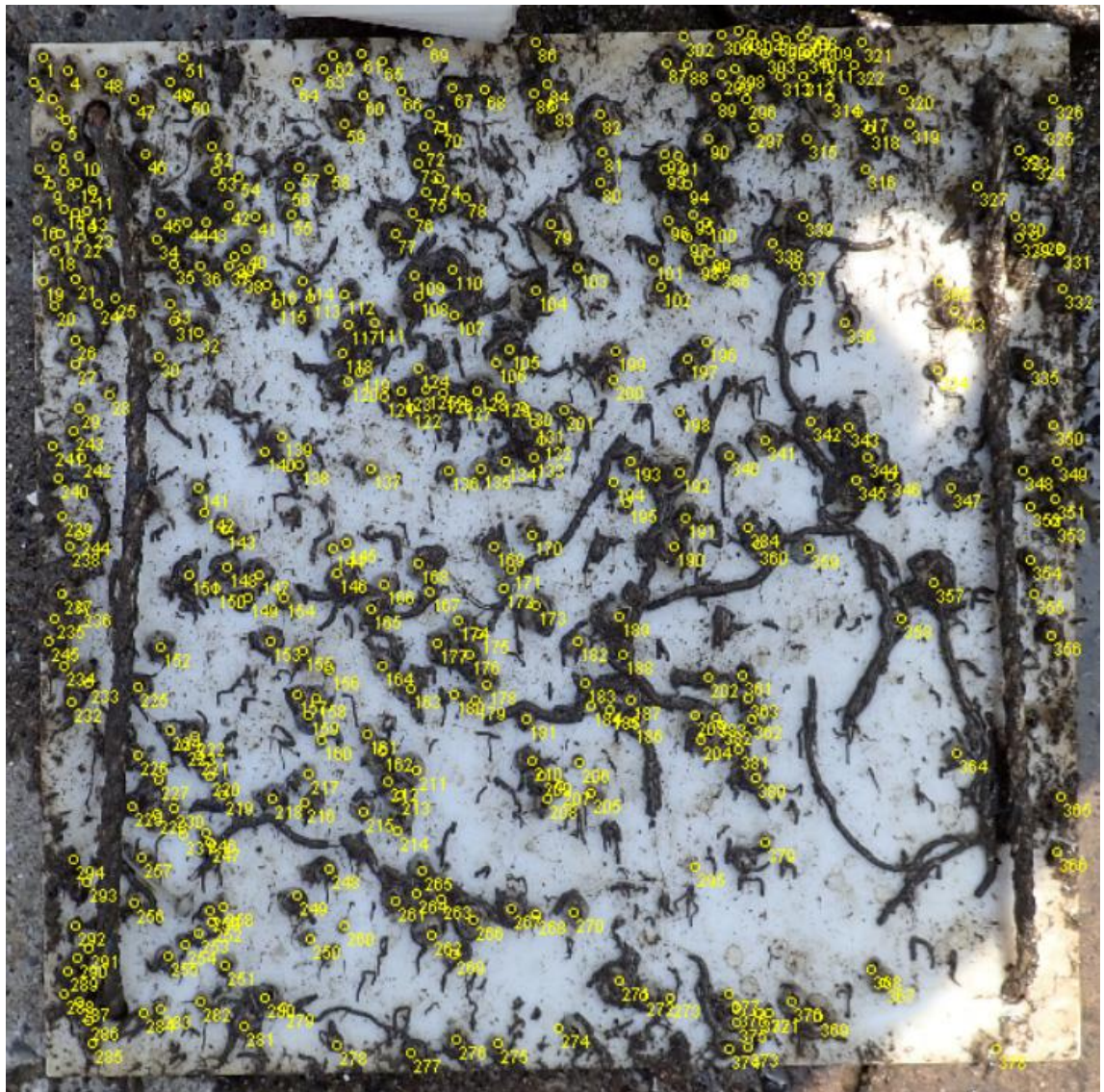

(10)

Supplement: Supplemental Information 6 [file peerj-10-14002-s006.pdf]
